# Pillar Modularity in **fsc** Topology Hybrid Ultramicroporous Materials Based upon Tetra(4-pyridyl)benzene

**DOI:** 10.1021/acs.cgd.2c00561

**Published:** 2022-08-19

**Authors:** Debobroto Sensharma, Benjamin H. Wilson, Naveen Kumar, Daniel J. O’Hearn, Michael J. Zaworotko

**Affiliations:** Department of Chemical Sciences, Bernal Institute, University of Limerick, Limerick V94 T9PX, Republic of Ireland

## Abstract

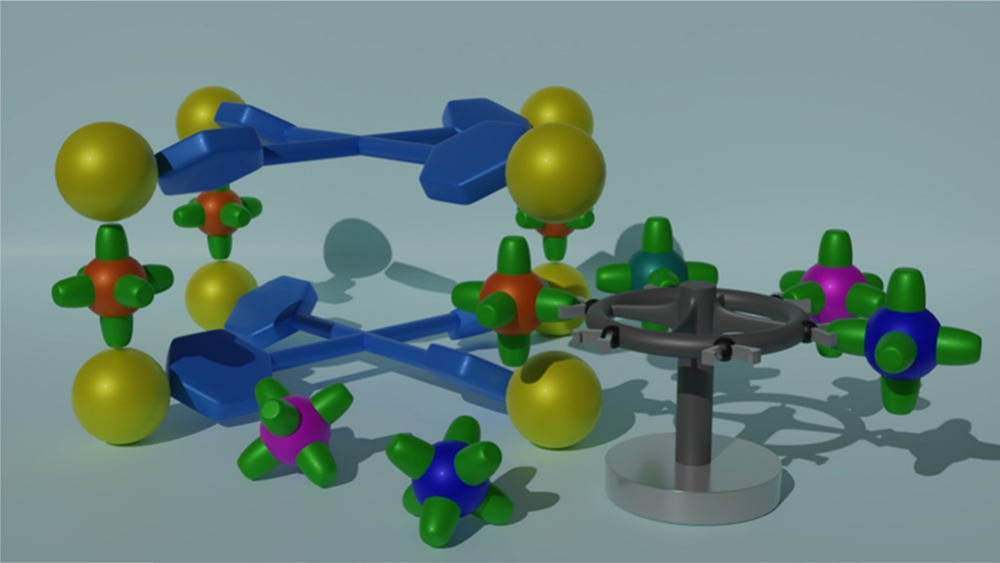

Hybrid ultramicroporous materials (HUMs) are porous coordination networks composed of combinations of organic and inorganic linker ligands with a pore diameter of <7 Å. Despite their benchmark gas sorption selectivity for several industrially relevant gas separations and their inherent modularity, the structural and compositional diversity of HUMs remains underexplored. In this contribution, we report a family of six HUMs (**SIFSIX-22-Zn**, **TIFSIX-6-Zn**, **SNFSIX-2-Zn**, **GEFSIX-4-Zn**, **ZRFSIX-3-Zn**, and **TAFSEVEN-1-Zn**) based on Zn metal centers and the tetratopic N-donor organic ligand tetra(4-pyridyl)benzene (**tepb**). The incorporation of fluorinated inorganic pillars (SiF_6_^2–^, TiF_6_^2–^, SnF_6_^2–^, GeF_6_^2–^, ZrF_6_^2–^, and TaF_7_^2–^, respectively) resulted in (4,6)-connected **fsc** topology as verified using single-crystal X-ray diffraction. Pure-component gas sorption studies with N_2_, CO_2_, C_2_H_2_, C_2_H_4_, and C_2_H_6_ revealed that the large voids and narrow pore windows common to all six HUMs can be leveraged to afford high C_2_H_2_ uptakes while retaining high ideal adsorbed solution theory (IAST) selectivities for industrially relevant gas mixtures: >10 for 1:99 C_2_H_2_/C_2_H_4_ and >5 for 1:1 C_2_H_2_/CO_2_. The approach taken, systematic variation of pillars with retention of structure, enables differences in selectivity to be attributed directly to the choice of the inorganic pillar. This study introduces **fsc** topology HUMs as a modular platform that is amenable to fine-tuning of structure and properties.

## Abstract 2

The inherent modularity of a family of hybrid ultramicroporous materials (HUMs) with **fsc** topology enables a systematic study of structure−property relationships through inorganic pillar substitution.

## Introduction

[Zn(4,4′-bipyridine)_2_(SiF_6_)]_*n*_ (**SIFSIX-1-Zn**), reported in 1995,^1^ is the prototypal hybrid coordination network (HCN) and is the parent of what is today a broad and growing platform of porous materials with a diverse range of topologies, pore sizes, pore chemistries, and properties.^2^ That both the pore size and pore chemistry of HCNs are amenable to fine-tuning through crystal engineering approaches is a consequence of their inherently modular nature, which comes from their typical compositions: a divalent metal ion node, neutral *N*-donor linker ligand, and inorganic anion pillar.^2^ The resulting hybrid coordination networks are thereby composed of geometrically simple components.^3^ The ready availability of *N*-donor linker ligands of varying lengths (*e.g.*, pyrazine, 2.8 Å; 4,4′-bipyridine, 7.1 Å; *N*,*N*′*-*di(4-pyridyl)-1,4,5,8-naphthalenediimide, 15.4 Å) and inorganic dianions that can serve as linkers that offer strong electrostatics (e.g., SiF_6_^2–^, GeF_6_^2–^, AlF_5_^2–^, NbOF_5_^2–^, MoO_4_^2–^, Cr_2_O_7_^2–^) has been exploited to fine-tune properties that are of relevance to several important industrial separation challenges.^4−11^ In particular, HCNs with pore diameters <7 Å (hybrid ultramicroporous materials, HUMs) exhibit selectivity values that are in some cases orders of magnitude greater than previous benchmark porous materials: **SIFSIX-18-Ni-β**, **NbOFFIVE-1-Ni**, and **TIFSIX-3-Ni** for direct air capture of CO_2_; **SIFSIX-14-Cu-i** for removal of acetylene from ethylene; and **CROFOUR-1-Ni** for Xe/Kr separation.^12−22^

Although crystal engineering has enabled systematic access to platforms of HUMs with exceptional properties, there is limited topological diversity among the HUMs reported thus far. The majority of HCNs, including HUMs, are **pcu** topology nets composed of octahedral metal centers linked by two ditopic *N*-donor linker ligands and a ditopic inorganic pillar anion. Among the non-**pcu** topology HCNs, only those that exhibit **mmo** topology have been studied systematically.^23^ Prior to the recent reports of **ZJU-280** (**SIFSIX-22-Cu**, [Cu(tepb)SiF_6_]) and **TIFSIX-Cu-TPB** (**TIFSIX-6-Cu**, [Cu(tepb)TiF_6_]), **fsc-2-SIFSIX** ([Cu_3_(4-(pyridin-4-yl)acrylic acid)_4_(SiF_6_)]), **CPM-131** ([(TPyP-Fe)Zn(SiF_6_)]), and its analogues ([(TPyP-M)Cu(NbOF_5_)], M = Zn, Fe, Ni) were the only HCNs with (4,6)-connected **fsc** topology, and their sorption properties were found to be driven by coordinatively unsaturated metal centers (UMCs) rather than electrostatics and tight binding sites.^24−28^ In **fsc-2-SIFSIX**, a bifunctional organic linker ligand allows for incorporation of both mononuclear Cu(II) and dinuclear {Cu_2_} paddle-wheel building blocks into the final structure.^28^**CPM-131** (and the related **fsx** net **CPM-132**) is constructed using a porphyrin-based metalloligand, and despite the challenges of tuning a porphyrin-based system, it exemplifies HCNs based on a polytopic (used herein to refer to connectivity >2) ligand.^25,26^ In 2016, Lusi et al. reported a family of HCNs based on the polytopic linker Tripp (2,4,6-tris(4-pyridyl)pyridine), **Tripp-Cu-MFSIX** ([[Cu_6_(Tripp)_8_](MF_6_)_3_(MF_6_)_3_]).^29^ These structures formed a partially bridged **pto**-type net but were not found to be permanently porous despite large solvent-accessible voids. A notable feature of this platform is that five distinct inorganic pillar dianions were incorporated into the same structure. More recently, Wu *et al.* reported an **ith-d** topology framework, **SIFSIX-Cu-TPA** ([Cu_3_(TPA)_4_(SiF_6_)_3_]), using a tritopic linker ligand, TPA (tri(pyridin-4-yl)amine).^30^ Our group recently reported the **fsc** frameworks **SIFSIX-22-Zn** and **SOFOUR-1-Zn**, both of which are based on the tepb linker.^31^

The modularity of a porous coordination network (PCN) can be expressed in terms of how many components can be varied independently. HCNs based on two-dimensional nets pillared by MFSIX to form **pcu** networks are highly modular, having three components that can be varied independently (organic linker ligand, inorganic anionic pillar ligand, and metal cation node).^2^ Additionally, there are cases in which interpenetration can also be controlled, for example, in **SIFSIX-2-Cu** and **SIFSIX-2-Cu-i**.^4^ The resulting drastic effect on the pore size and pore chemistry that results from interpenetration (and therefore properties) offers a fourth variable by which such a platform can be modulated. This level of modularity (four components) is only met or surpassed by platforms that combine mixed linkers and/or extra-framework anions/cations. Most other well-known PCNs, such as those based on oxo-clusters and carboxylate linkers, offer just one or two modular components, limiting the scope of related materials that can be generated and, therefore, the extent to which properties can be “fine-tuned”.

A crystal engineering approach predominantly based on ditopic linker ligands means that most HCNs exhibit nearly cylindrical one-dimensional channels with a high density of tight binding sites to drive their sorption properties. The design of HCNs based on polytopic ligands offers the possibility of new types of channel architectures and new structure–property relationships. In their recent work on **ZJU-280 (SIFSIX-22-Cu)**, Qian and co-workers reported a HUM that is composed of a tetratopic linker ligand in place of the more commonly used ditopic linkers, presenting an opportunity for the development of a new HUM platform for the study of structure–property relationships.^24^ We recently reported **SIFSIX-22-Zn** and **SOFOUR-1-Zn** using the same tetrapyridyl linker and SiF_6_^2–^ or SO_4_^2–^ pillars, respectively.^31^ In the present work, we report a crystal engineering study of **fsc** HUMs involving substitution of the inorganic pillars in [Zn(tepb)SiF_6_] (**SIFSIX-22-Zn**) to afford an additional five members of this platform: [Zn(tepb)TiF_6_] (**TIFSIX-6-Zn)**, [Zn(tepb)SnF_6_] (**SNFSIX-2-Zn**), [Zn(tepb)GeF_6_] (**GEFSIX-4-Zn**), [Zn(tepb)ZrF_6_] (**ZRFSIX-3-Zn**), and [Zn(tepb)TaF_7_] (**TAFSEVEN-1-Zn**).

## Experimental Section

### Materials and Methods

All reagents and solvents were used as received from vendors. ^1^H NMR spectroscopy was performed using a JEOL ECX400 spectrometer operating at 400 MHz. Thermal gravimetric analysis (TGA) was performed using a TA Q50 analyzer with a ramp rate of 10.00 °C/min from 25 to 500 °C and nitrogen gas flow of 40 mL/min. Powder X-ray diffraction (PXRD) diffractograms were recorded using a PANalytical X’Pert operated at 40 kV and 40 mA and CuK_α_ radiation (λ_α_ = 1.540598 Å) was used for diffraction experiments. Incident beam optics included the Fixed Divergence slit with antiscatter slit PreFIX module, with a 1/8° divergence slit and a 1/4° antiscatter slit, as well as a 10 mm fixed incident beam mask and a Ni-β filter. Data were collected from 5°–40° (2θ) with a step-size of 0.0131303° and a scan time of 30 s per step.

### Synthesis of 1,2,4,5-Tetra(4-pyridyl)benzene, tepb

1,2,4,5-Tetra(4-pyridyl)benzene (**tepb**) was synthesized according to the procedure reported by Chang and Wang.^32^ Fe(NO_3_)_3_·9H_2_O (0.161 g, 0.4 mmol), H_3_PO_4_ (0.6 mL, 9 mmol), 1,3-bis(4-pyridyl)propane (1.268 g, 6.4 mmol), oxalic acid dihydrate (0.151 g, 1.2 mmol), and water (2 mL) were combined in a Teflon-lined pressure vessel and heated at 180 °C for 48 h. Needle crystals of tepb were manually removed, washed with cold methanol, and dried (yield: *ca.* 50%). ^1^H NMR (400 MHz, DMSO-d_6_): δ = 8.48 (8H, d), 7.61 (2H, s), 7.24 (8H, d).

### Synthesis of [Zn(tepb)SiF_6_], **SIFSIX-22-Zn**

A solution of ZnSiF_6_·6H_2_O (1.0 mg, 0.003 mmol) in 0.4 mL of methanol was put in a narrow glass tube. Methanol (0.2 mL) was carefully layered over this solution to act as a buffer layer before a solution of **tepb** (1.2 mg, 0.003 mmol) in 0.4 mL methanol was layered over the buffer layer. The tube was left undisturbed for 5 days, at which point colorless block crystals of [Zn(tepb)SiF_6_]·*x*MeOH (as-synthesized **SIFSIX-22-Zn**) were obtained. A larger quantity of **SIFSIX-22-Zn** was prepared as follows: ZnSiF_6_·6H_2_O (41.2 mg, 0.13 mmol) was added to a solution of **tepb** (77.2 mg, 0.20 mmol) in 16 mL methanol and stirred at room temperature overnight. [Zn(tepb)SiF_6_]·*x*MeOH was obtained as a white microcrystalline powder which was isolated by filtration before being washed with methanol and air dried. Yield: 43.0 mg, 56%. CHN analysis calculated for C_26_H_26_F_6_N_4_O_4_SiZn (including four interstitial water molecules): C 46.89%, H 3.94%, N 8.41%; experimental: C 46.72%, H 3.12%, N 8.27%.

### Synthesis of [Zn(tepb)TiF_6_], **TIFSIX-6-Zn**

A solution of Zn(NO_3_)_2_·6H_2_O (0.89 mg, 0.003 mmol) and (NH_4_)_2_TiF_6_ (0.59 mg, 0.003 mmol) in 0.2 mL of water was placed in a narrow glass tube. 1:1 methanol/water (0.4 mL) was carefully layered over this solution to act as a buffer layer before a solution of **tepb** (1.2 mg, 0.003 mmol) in 0.4 mL methanol was layered over the buffer layer, and the tube was left undisturbed for 3 days, at which point colorless block crystals of [Zn(tepb)TiF_6_]·*x*MeOH (as-synthesized **TIFSIX-6-Zn**) were obtained. A bulk sample of **TIFSIX-6-Zn** was prepared as follows: Zn(NO_3_)_2_·6H_2_O (77.3 mg, 0.26 mmol) and (NH_4_)_2_TiF_6_ (51.5 mg, 0.26 mmol) in 1.0 mL of water was added to a solution of **tepb** (154.4 mg, 0.4 mmol) in 30 mL methanol and stirred at room temperature overnight. [Zn(tepb)TiF_6_]·*x*MeOH was obtained as a white microcrystalline powder, isolated by filtration, washed with methanol, and air-dried. Yield: 96 mg, 60%. CHN analysis calculated for C_26_H_28_F_6_N_4_O_5_TiZn (including five interstitial water molecules): C 44.37%, H 4.01%, N 7.96%; experimental: C 44.37%, H 3.41%, N 8.11%.

### Synthesis of [Zn(tepb)GeF_6_], **GEFSIX-4-Zn**

A solution of Zn(NO_3_)_2_·6H_2_O (0.89 mg, 0.003 mmol) and (NH_4_)_2_GeF_6_ (0.66 mg, 0.003 mmol) in 0.2 mL of water was placed in a narrow glass tube. 1:1 methanol/water (0.4 mL) was carefully layered over this solution to act as a buffer layer. Finally, a solution of **tepb** (1.2 mg, 0.003 mmol) in 0.4 mL methanol was layered over the buffer layer, and the tube was left undisturbed for 3 days. Colorless block crystals of [Zn(tepb)GeF_6_]·*x*MeOH (as-synthesized **GEFSIX-4-Zn**) were thereby obtained. A bulk sample of **GEFSIX-4-Zn** was prepared as follows: Zn(NO_3_)_2_·6H_2_O (77.3 mg, 0.26 mmol) and (NH_4_)_2_GeF_6_ (57.9 mg, 0.26 mmol) in 1.0 mL of water was added to a solution of **tepb** (154.4 mg, 0.4 mmol) in 30 mL methanol and stirred at room temperature overnight. [Zn(tepb)GeF_6_]·*x*MeOH was obtained as a white microcrystalline powder, which was isolated by filtration, washed with methanol, and air-dried. Yield: 108 mg, 65%. CHN analysis calculated for C_26_H_26_F_6_N_4_O_4_GeZn (including four interstitial water molecules): C 43.95%, H 3.69%, N 7.87%; experimental: C 43.95%, H 3.24%, N 7.89%.

### Synthesis of [Zn(tepb)SnF_6_], **SNFSIX-2-Zn**

A solution of Zn(NO_3_)_2_·6H_2_O (0.89 mg, 0.003 mmol) and (NH_4_)_2_SnF_6_ (0.80 mg, 0.003 mmol) in 0.2 mL of water was placed in a narrow glass tube. 1:1 methanol/water (0.4 mL) was carefully layered over this solution to act as a buffer layer. Finally, a solution of **tepb** (1.2 mg, 0.003 mmol) in 0.4 mL methanol was layered over the buffer layer, and the tube was left undisturbed for 3 days. Colorless block crystals of [Zn(tepb)SnF_6_]·*x*MeOH (as-synthesized **SNFSIX-2-Zn**) were thereby obtained. A bulk sample of **SNFSIX-2-Zn** was prepared as follows: a solution Zn(NO_3_)_2_·6H_2_O (77.3 mg, 0.26 mmol) and (NH_4_)_2_SnF_6_ (69.4 mg, 0.26 mmol) in 1.0 mL of water was added to a solution of **tepb** (154.4 mg, 0.4 mmol) in 30 mL methanol and stirred at room temperature overnight. [Zn(tepb)SnF_6_]·*x*MeOH was obtained as a white microcrystalline powder, which was isolated by filtration, washed with methanol, and air-dried. Yield: 119 mg, 67%. CHN analysis calculated for C_26_H_34_F_6_N_4_O_8_SnZn (including eight interstitial water molecules): C 37.69%, H 4.14%, N 6.76%; experimental: C 37.57%, H 3.20%, N 6.75%.

### Synthesis of [Zn(tepb)ZrF_6_], **ZRFSIX-3-Zn**

A solution of Zn(NO_3_)_2_·6H_2_O (0.89 mg, 0.003 mmol) and K_2_ZrF_6_ (0.85 mg, 0.003 mmol) in 0.2 mL of water was placed in a narrow glass tube. 1:1 methanol/water (0.4 mL) was carefully layered over this solution to act as a buffer layer. Finally, a solution of **tepb** (1.2 mg, 0.003 mmol) in 0.4 mL methanol was layered over the buffer layer, and the tube was left undisturbed for 3 days. Colorless block crystals of [Zn(tepb)ZrF_6_]·*x*MeOH (as-synthesized **ZRFSIX-3-Zn**) were thereby obtained. A bulk sample of **ZRFSIX-3-Zn** was prepared as follows: A solution Zn(NO_3_)_2_·6H_2_O (77.3 mg, 0.26 mmol) and K_2_ZrF_6_ (73.7 mg, 0.26 mmol) in 10.0 mL of water was added to a solution of **tepb** (154.4 mg, 0.4 mmol) in 30 mL methanol and stirred at room temperature overnight. [Zn(tepb)ZrF_6_]·*x*MeOH was obtained as a white microcrystalline powder, which was isolated by filtration, washed with methanol, and air-dried. Yield: 116 mg, 68%. CHN analysis calculated for C_26_H_28_F_6_N_4_O_5_ZrZn (including five interstitial water molecules): C 41.80%, H 3.78%, N 7.50%; experimental: C 41.92%, H 2.91%, N 7.82%.

### Synthesis of [Zn(tepb)TaF_7_], **TAFSEVEN-1-Zn**

A solution of Zn(NO_3_)_2_·6H_2_O (0.89 mg, 0.003 mmol) and (NH_4_)_2_TaF_7_ (1.05 mg, 0.003 mmol) in 0.2 mL of water was placed in a narrow glass tube. 1:1 methanol/water (0.4 mL) was carefully layered over this solution to act as a buffer layer. Finally, a solution of **tepb** (1.2 mg, 0.003 mmol) in 0.4 mL methanol was layered over the buffer layer, and the tube was left undisturbed for 3 days. Colorless block crystals of [Zn(tepb)TaF_7_]·*x*MeOH (as-synthesized **TAFSEVEN-1-Zn**) were thereby obtained. A bulk sample of **TAFSEVEN-1-Zn** was prepared as follows: a solution Zn(NO_3_)_2_·6H_2_O (77.3 mg, 0.26 mmol) and (NH_4_)_2_TaF_7_ (91.0 mg, 0.26 mmol) in 1 mL of water was added to a solution of **tepb** (154.4 mg, 0.4 mmol) in 30 mL methanol and stirred at room temperature overnight. [Zn(tepb)TaF_7_]·*x*MeOH was obtained as a white microcrystalline powder, which was isolated by filtration, washed with methanol, and air-dried. Yield: 113 mg, 57%. CHN analysis calculated for C_26_H_22_F_6_N_4_O_2_TaZn (including two interstitial water molecules): C 38.95%, H 2.77%, N 6.99%; experimental: C 38.81%, H 2.25%, N 7.05%.

### X-ray Crystallography

Single-crystal X-ray crystallographic data were collected at 298 K on a Bruker D8 Quest diffractometer equipped with a CuKα microfocus source (λ = 1.5406 Å) and Photon 100 detector. Temperature was controlled using a nitrogen flow from Oxford Cryosystems. Data was indexed, integrated and scaled in APEX3.^33^ Absorption correction was performed by the multi-scan method using SADABS.^34^ Space group determination was performed simultaneously with structure solution using the intrinsic phasing method (SHELXT),^35^ and the solution was refined on *F*^2^ using SHELXL^36^ nonlinear least squares implemented in Olex2 v1.2.10.^37^ All nonhydrogen atoms were refined anisotropically and hydrogen atoms bonded to carbon atoms were added at calculated positions and refined using a riding model. Disordered solvents were found in the cavity of all structures. Some of this electron density could be modeled as methanol molecules with partial occupancy; however, refinement was unsatisfactory, and the solvent atomic displacement parameters were unreasonable. The PLATON SQUEEZE^38^ routine was performed to account for the electron density of unmodelled solvents, resulting in a more satisfactory refinement. The crystal structure CIF files have been deposited in the Cambridge Crystallographic Data Centre (CCDC: 2151309–2151313).

### Gas Sorption Measurements

For gas sorption experiments, ultra-high purity gases were used as received from BOC Gases Ireland: He (99.999%), CO_2_ (99.995%), C_2_H_2_ (98.5%), N_2_ (99.998%), C_2_H_4_ (99.92%), and C_2_H_6_ (99.0%). Adsorption isotherm experiments (up to 1 bar) for 195 K CO_2_ were performed on a Micromeritics Tristar II 3030. A Micromeritics 3Flex surface area and pore size analyzer 3500 was used for collecting the 273 and 298 K sorption isotherms for all gases. The low temperature of 195 K was maintained using a dry ice-acetone mixture. Bath temperatures of 273 and 298 K were precisely controlled with a Julabo ME (v.2) recirculating control system containing a mixture of ethylene glycol and water. Prior to experiments, **SIFSIX-22-Zn**, **TIFSIX-6-Zn**, **GEFSIX-4-Zn**, **SNFSIX-2-Zn**, **ZRFSIX-3-Zn,** and **TAFSEVEN-1-Zn** were activated on a Smart VacPrep using dynamic vacuum and heating for 24 h at 333 K.

## Results and Discussion

### Structural Description

**Figure 1:**
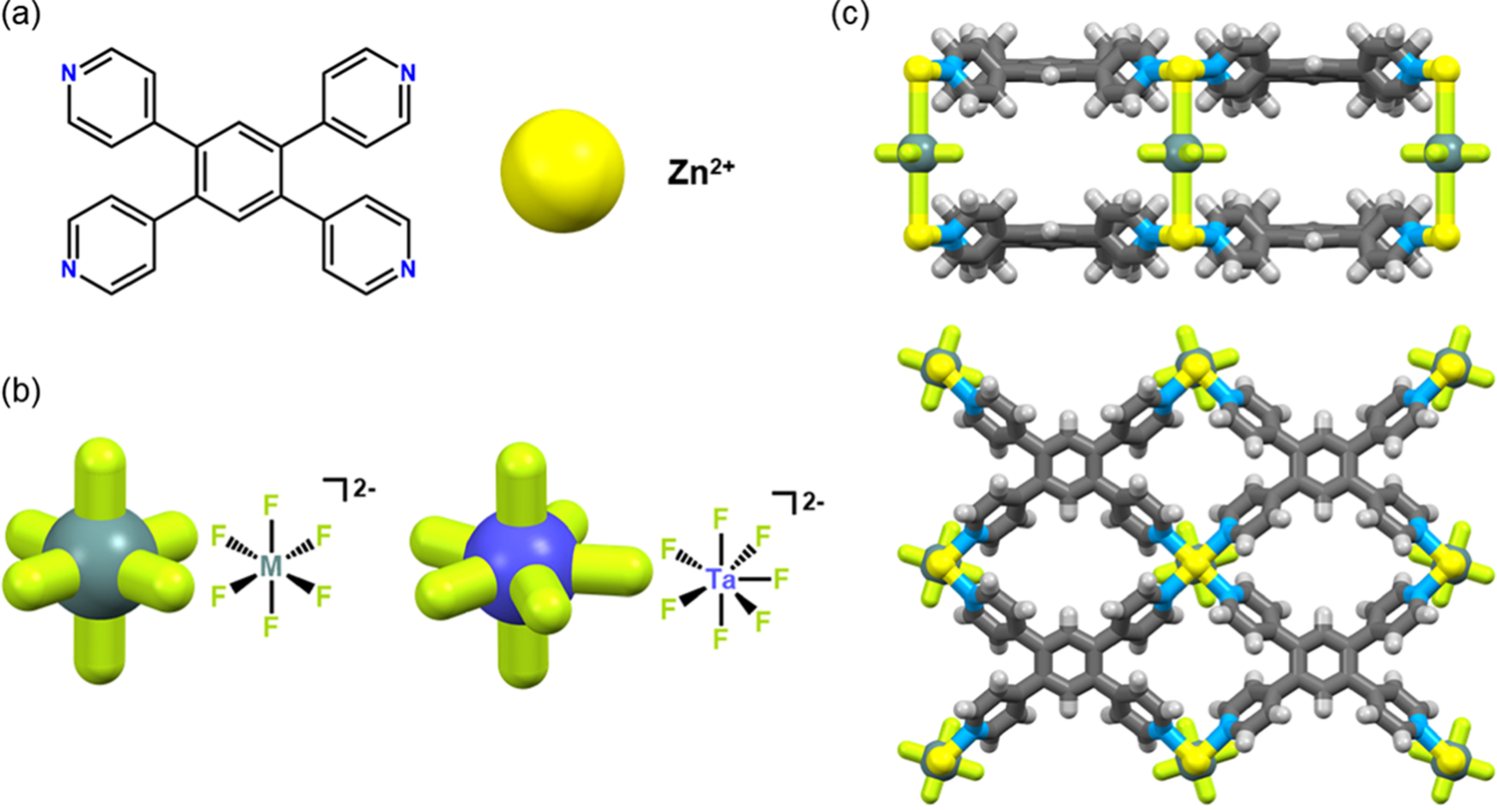
Representations of (a) tetra(4-pyridyl)benzene (tepb) ligand and Zn^2+^ metal center; (b) **MFSIX** and **TAFSEVEN** pillars; and (c) **fsc** network [Zn(tepb)MF_6_] viewed along the *c* (above) and *a* (below) crystallographic axes.

**TIFSIX-6-Zn** crystallized in the centrosymmetric orthorhombic space group *Pmma*, **SIFSIX-22-Zn**, **GEFSIX-4-Zn**, **SNFSIX-2-Zn**, and **TAFSEVEN-1-Zn** crystallized in the centrosymmetric orthorhombic space group *Cmma* while **ZRFSIX-3-Zn** crystallized in the centrosymmetric orthorhombic space group *Pmmm*. All six structures are comprised as expected: octahedral Zn^2+^ ions coordinated to four pyridyl moieties of **tepb** ligands in their equatorial positions and bridging MF_6_^2–^ (M = Si(IV), Ti(IV), Ge(IV), Sn(IV), and Zr(IV)) or TaF_7_^2–^ anions in their axial positions. The Zn^2+^ ions and **tepb** ligands formed two-dimensional layers pillared by the MF_6_^2–^ or TaF_7_^2–^ anions to generate three-dimensional 4,6-connected **fsc** topology networks (Figure 1).

The Zn–F distance in **TAFSEVEN-1-Zn** is 2.040(2) Å, which lies within the lower quintile (2.061 Å) of the mean distance of 2.108 Å (st. dev. = 0.059 Å) as per the Cambridge Structural Database^39^ (CSD, v. 5.41 + 3 updates, see the SI for search parameters), while the Zn–F distances for **SIFSIX-22-Zn**, **TIFSIX-6-Zn**, **GEFSIX-4-Zn**, **SNFSIX-2-Zn,** and **ZRFSIX-3-Zn** lie in the second quintile (2.092 Å). The **TAFSEVEN-1-Zn** Zn–N distance range of 2.176(3) Å lies within the upper quintile (2.157 Å) of the mean distance of 2.138 Å (st. dev. = 0.020 Å) as per the same search query. Zn–N distances for **GEFSIX-4-Zn** and **SNFSIX-2-Zn** lie in the third quintile (2.145 Å) while the Zn–N distances for **SIFSIX-22-Zn**, **TIFSIX-6-Zn,** and **ZRFSIX-3-Zn** lie in the fourth quintile (2.157 Å).

**Figure 2:**
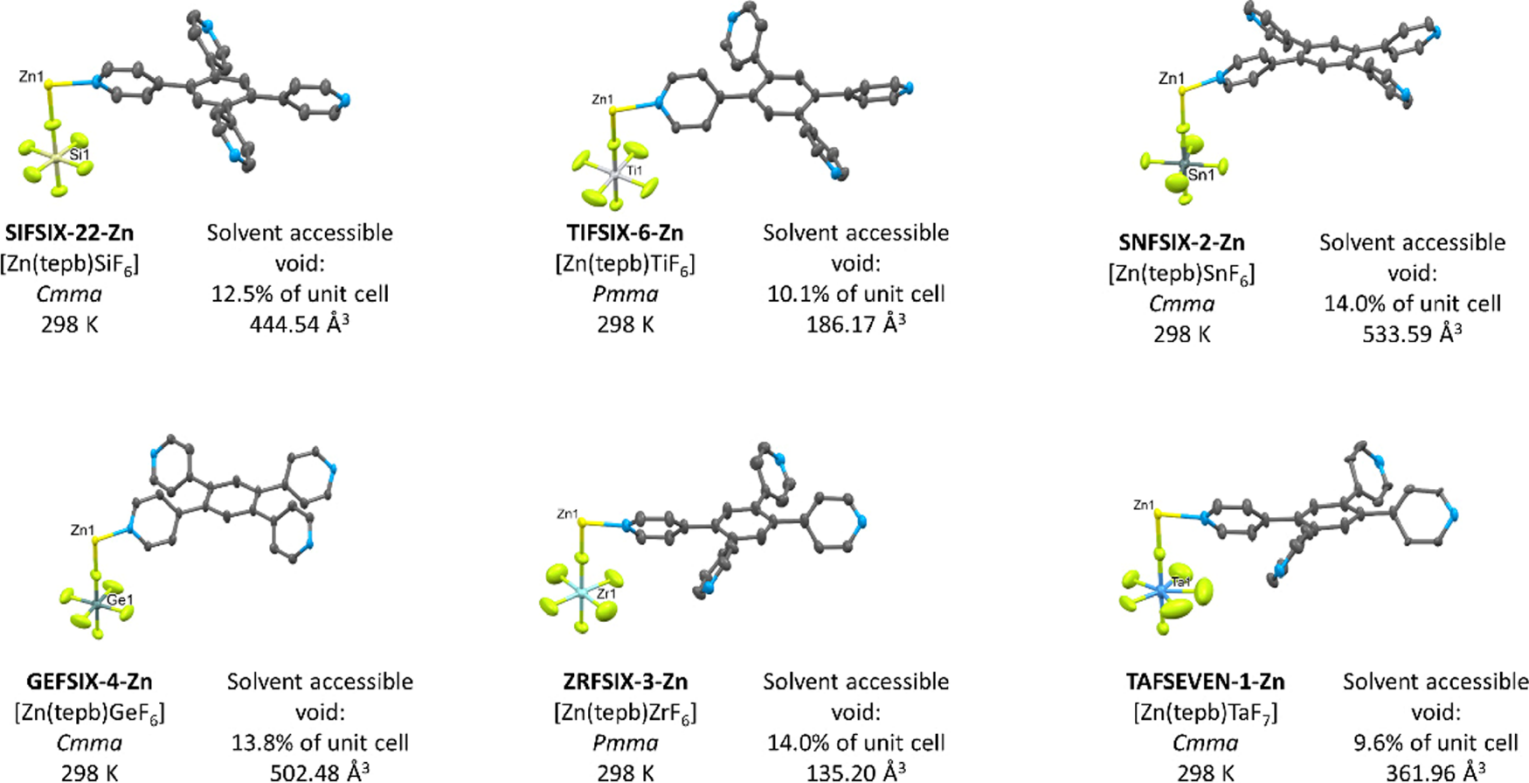
Formula units in **SIFSIX-22-Zn**, **TIFSIX-6-Zn**, **SNFSIX-2-Zn**, **GEFSIX-4-Zn**, **ZRFSIX-3-Zn**, and **TAFSEVEN-1-Zn**. Thermal ellipsoids are shown at a probability level of 50%.

The pyridine rings of the **tepb** ligand adopt a propeller-type arrangement about the central benzene ring with rings *para* to each other being co-planar in **TIFSIX-6-Zn**, **GEFSIX-4-Zn**, **SNFSIX-2-Zn**, **ZRFSIX-3-Zn**, and **TAFSEVEN-1-Zn** (Figure 2). The torsion angles of the pyridine ring about the Zn^2+^ ions are given in Figures S1–S4 and range from 52.3(2)° to 70.11(5)°. The pyridyl rings are arranged in a propeller-like conformation about the zinc(II) ion. The two-dimensional Zn^2+^-**tepb** layers are pillared by the MF_6_^2–^ and TaF_7_^2–^ pillars such that the central aromatic rings of the **tepb** ligands are eclipsed and coplanar when viewed down the crystallographic *a*-axis for **SIFSIX-22-Zn**, **GEFSIX-4-Zn**, **SNFSIX-2-Zn**, **ZRFSIX-3-Zn**, and **TAFSEVEN-1-Zn** and the crystallographic *b*-axis for **TIFSIX-6-Zn**. In the case of **TIFSIX-6-Zn** and **ZRFSIX-3-Zn**, the fluorine atoms are modeled as disordered over two positions with each position being eclipsed with the fluorine atoms above and below the plane. For **SIFSIX-22-Zn**, **GEFSIX-4-Zn**, **SNFSIX-2-Zn**, and **TAFSEVEN-1-Zn**, the equatorial fluorine atoms of the MF_*n*_^2–^ pillar are not eclipsed with **GEFSIX-4-Zn** having the largest pillar rotation angle of 55.0(2)°. In the case of **SNFSIX-2-Zn**, the equatorial fluorine atoms of the SnF_6_^2–^ pillar are modeled as disordered over two positions with each position exhibiting noticeably different pillar rotation angles: 4.9(5)° and 23.0(5)°. In the two-dimensional zinc(II)-**tepb** layers, there are two distinct windows (Figure S6), the sizes of which are roughly consistent across all six compounds as the window geometry is independent of the anionic pillar: narrow, square windows where the pyridyl rings are *ortho* relative to one another; larger rectangular windows when pyridyl rings are *meta* relative to one another.

**Figure 3:**
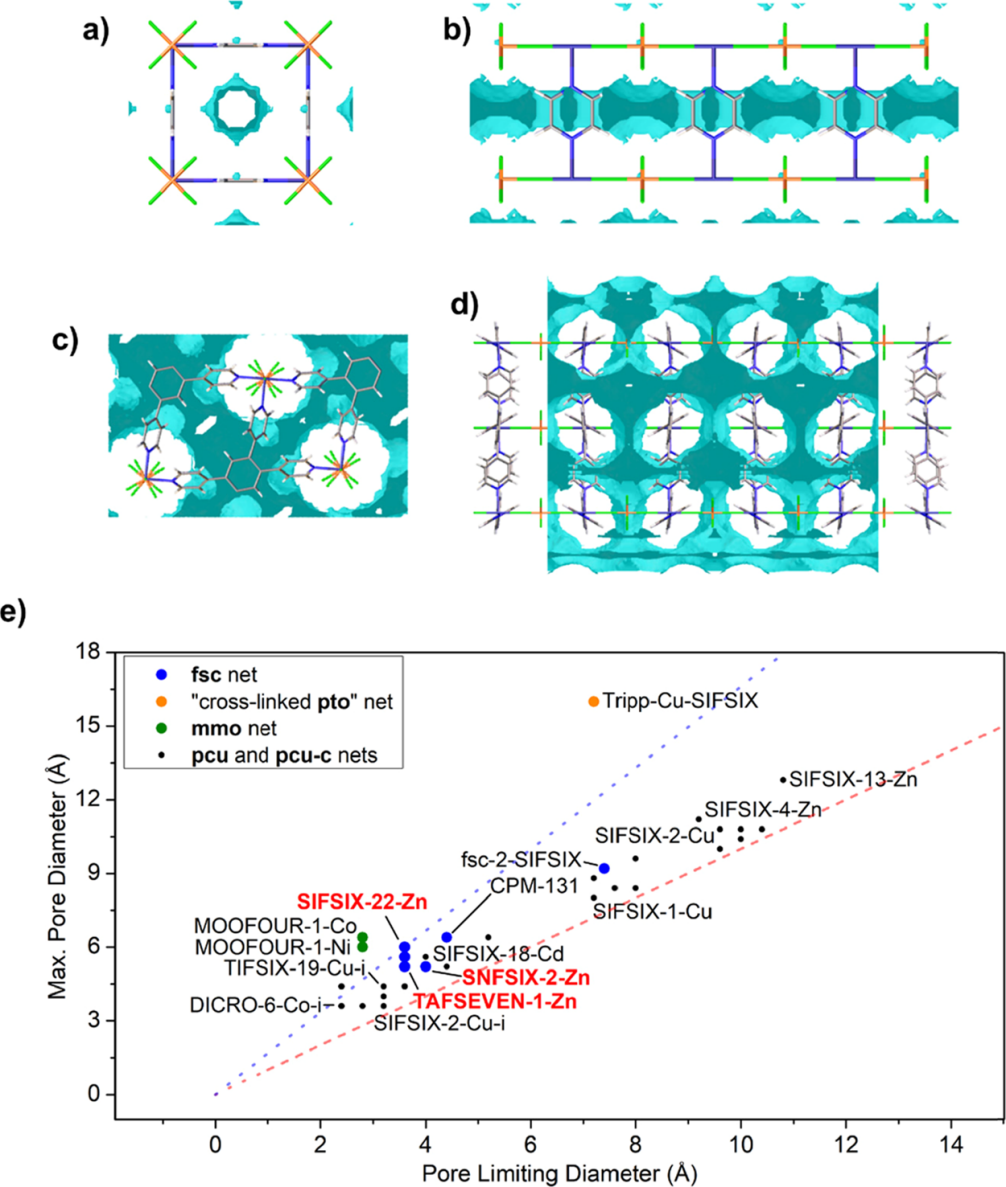
Accessible void surface calculated for **SIFSIX-3-Zn** viewed along the crystallographic (a) *a*- and (b) *c*-directions. Representations of the accessible void surface calculated for **SIFSIX-22-Zn** viewed along the crystallographic (c) *a*- and (d) *c*-directions. (e) Plot of crystallographically determined maximum and limiting pore diameters in representative HCN materials (red dashed line: idealized cylindrical pores; blue dashed line: the ratio of maximum pore diameter to pore limiting diameter in **SIFSIX-22-Zn**; see the SI for tabulation).

Pore structures were calculated from the crystallographic data of **SIFSIX-22-Zn**, **TIFSIX-6-Zn**, **GEFSIX-4-Zn**, **SNFSIX-2-Zn**, **ZRFSIX-3-Zn**, and **TAFSEVEN-1-Zn** (Poreblazer v4.0, Tables S7, S8). The pore volume per formula unit ranges from 90.49 Å^3^ for **TAFSEVEN-1-Zn** to 135.20 Å^3^ for **ZRFSIX-3-Zn**. The maximum pore diameters vary from 4.71 to 5.92 Å (Poreblazer v4.0), whereas the limiting pore diameters range from 3.18 to 3.72 Å.^40^ The constricted pore region is a result of the narrow pore windows occurring in the Zn^2+^-**tepb** layers while the larger pore cavity exists between these two-dimensional layers. This degree of pore constriction is high compared to other HCNs such as **mmo** topology networks and **Tripp-Cu-SIFSIX** despite their unique network architectures.^23,29^ The highly constricted pores present in **SIFSIX-22-Zn**, **TIFSIX-6-Zn**, **GEFSIX-4-Zn**, **SNFSIX-2-Zn**, **ZRFSIX-3-Zn**, and **TAFSEVEN-1-Zn** are comparable to **SIFSIX-18-M** and **DICRO-6-Co-i** among the **pcu** topology HCNs which feature constricted pore environments (Figure 3). For both of the aforementioned materials, the pore constriction arises from the distortion of the one-dimensional metal-pillar-metal chain from a linear to zig-zag arrangement. In **SIFSIX-18-M**, this is due to the shape of the ligand, whereas in **DICRO-6-Co-i** it results from the nonlinear geometry of the pillar.^13,41^ In contrast, the constriction of pores in **SIFSIX-22-Zn**, **TIFSIX-6-Zn**, **GEFSIX-4-Zn**, **SNFSIX-2-Zn**, **ZRFSIX-3-Zn**, and **TAFSEVEN-1-Zn** arises solely from the dimensions of the Zn^2+^-**tepb** layers.

### Characterization

Bulk samples of each compound were synthesized by room-temperature slurry methods and characterized by PXRD and TGA (Figures S9–S14). PXRD patterns collected after immersion under MeOH for 1 week revealed that all samples had retained their crystal structures. PXRD patterns collected after exposure to accelerated stability test conditions at 75% R.H. and 40 °C after 1 day and 1 week revealed that **SIFSIX-22-Zn** underwent hydrolysis and an associated phase change within 1 day, while **GEFSIX-4-Zn** underwent a phase change between 1 day and 1 week. No change was observed in the PXRD patterns of **TIFSIX-6-Zn**, **SNFSIX-2-Zn**, **ZRFSIX-3-Zn,** and **TAFSEVEN-1-Zn** after 1 week, indicating that these pillars provide enhanced hydrolytic stability.

### Gas Sorption

**Figure 4:**
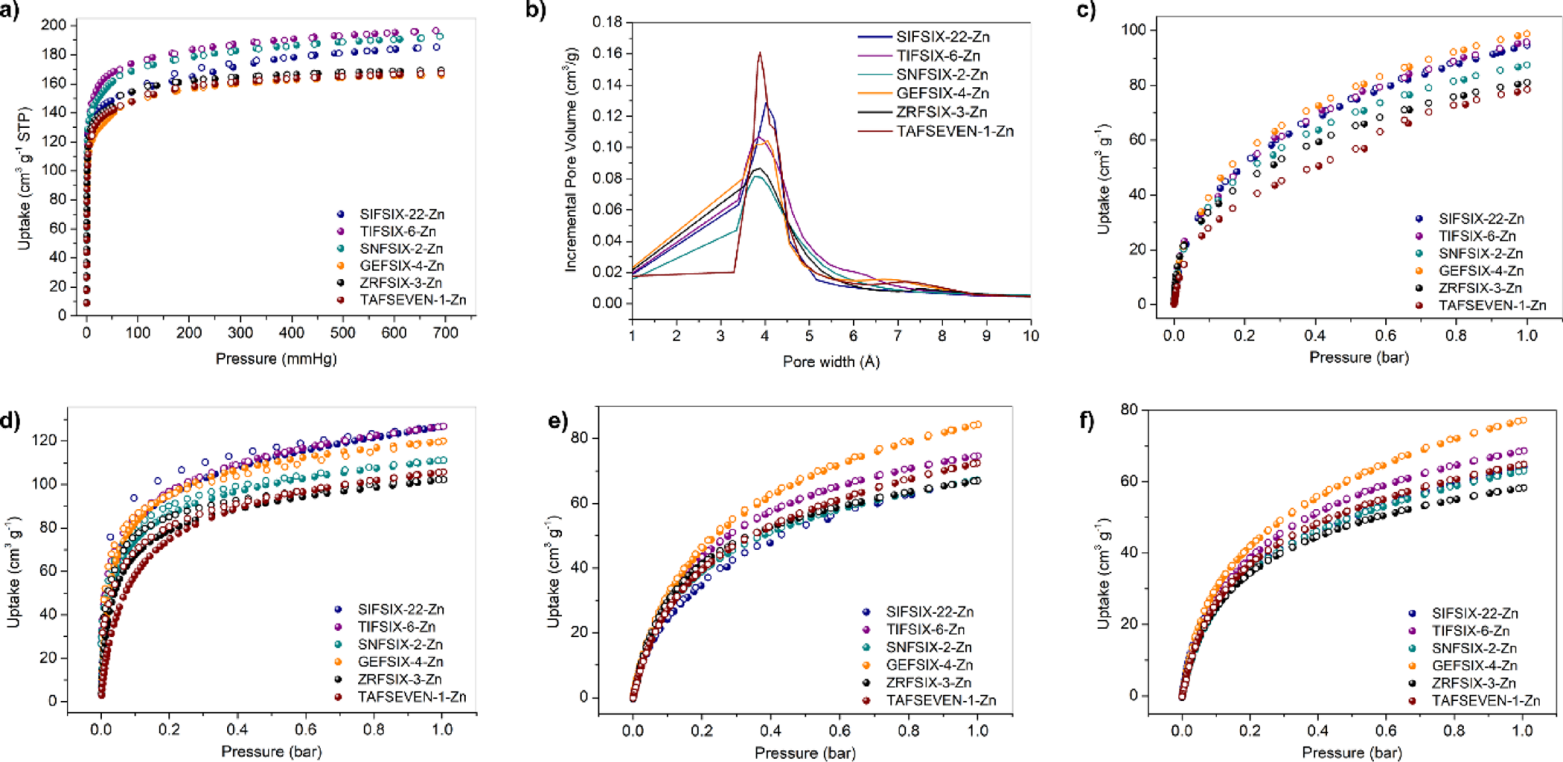
(a) 195 K CO_2_ sorption isotherms on **SIFSIX-22-Zn**, **TIFSIX-6-Zn**, **GEFSIX-4-Zn**, **SNFSIX-2-Zn**, **ZRFSIX-3-Zn**, and **TAFSEVEN-1-Zn**; (b) Horvath–Kawazoe pore-size distribution plots obtained using 195 K CO_2_ isotherms. 298 K sorption isotherms of (c) CO_2_, (d) C_2_H_2_, (e) C_2_H_4_, and (f) C_2_H_6_ on **SIFSIX-22-Zn**, **TIFSIX-6-Zn**, **GEFSIX-4-Zn**, **SNFSIX-2-Zn**, **ZRFSIX-3-Zn**, and **TAFSEVEN-1-Zn**.

After activating as-synthesized bulk samples of each HUM, CO_2_ sorption isotherms were measured at 195 K to determine their textural characteristics. In all six materials, type I isotherms with steep uptake at low pressure were observed with saturation uptakes of 150–200 cm^3^ g^–1^ (Figure 4a). Brunauer–Emmett–Teller (BET) surface areas of 387.2, 396.8, 700.0, 615.4, 625.9, and 627.5 m^2^ g^–1^ were determined for **SIFSIX-22-Zn**, **TIFSIX-6-Zn**, **GEFSIX-4-Zn**, **SNFSIX-2-Zn**, **ZRFSIX-3-Zn,** and **TAFSEVEN-1-Zn**, respectively. The Horvath–Kawazoe plots obtained from 195 K CO_2_ data revealed a narrow range of maximum pore widths between 3.7 and 4.1 Å (Figure 4b). These values experimentally confirm the categorization of these materials as ultramicroporous and lie within the range of crystallographically determined pore dimensions (Tables S7–S9). N_2_ sorption isotherms were also collected and afforded BET surface area values of 414.3, 478.1, 925.1, 798.1, 1062.0, and 652.9 m^2^ g^–1^ for **SIFSIX-22-Zn**, **TIFSIX-6-Zn**, **GEFSIX-4-Zn**, **SNFSIX-2-Zn**, **ZRFSIX-3-Zn,** and **TAFSEVEN-1-Zn**, respectively (Figures S16–S21). The variations between saturation CO_2_ and N_2_ uptakes are attributed to the differing sizes and quadrupole moments of N_2_ and CO_2_ and their interactions with the differing electrostatics of the surfaces of each adsorbent.^42^

Following these observations, we investigated the room-temperature sorption properties of each HUM toward CO_2_ and C2 hydrocarbons. The CO_2_, C_2_H_2_, C_2_H_4,_ and C_2_H_6_ sorption isotherms for **SIFSIX-22-Zn**, **TIFSIX-6-Zn**, **GEFSIX-4-Zn**, **SNFSIX-2-Zn**, and **ZRFSIX-3-Zn** are well-defined Langmuir-type profiles (Figure 4c–f). PXRD data collected after sorption experiments support the apparent reversibility of the isotherms and no loss of crystallinity was observed (Figures S9–S14). CO_2_ uptakes at 298 K varied from a maximum of 99 cm^3^ g^–1^ in **GEFSIX-4-Zn** to a minimum of 81 cm^3^ g^–1^ in **ZRFSIX-3-Zn**. Similarly, 298 K C_2_H_2_ uptakes ranged from 127 cm^3^ g^–1^ in **TIFSIX-6-Zn** to 102 cm^3^ g^–1^ in **ZRFSIX-3-Zn**. C_2_H_4_ and C_2_H_6_ isotherms exhibited similar profiles, with lower uptakes overall *vs.* C_2_H_2_. The only deviation from ideal Langmuir-type profiles was seen in the CO_2_ isotherm of **TAFSEVEN-1-Zn**, in which a minor inflection occurred at ca. 0.55 bar and 298 K. We attribute this anomaly to the five equatorial fluorides in the TaF_7_^2–^ pillar leading to a distinct electrostatic distribution vs MF_6_^2–^ pillars, thereby impacting sorption through F···H_Ar_ contacts to the **tepb** ligands by altering ligand conformation and pore dimensions.

**Figure 5:**
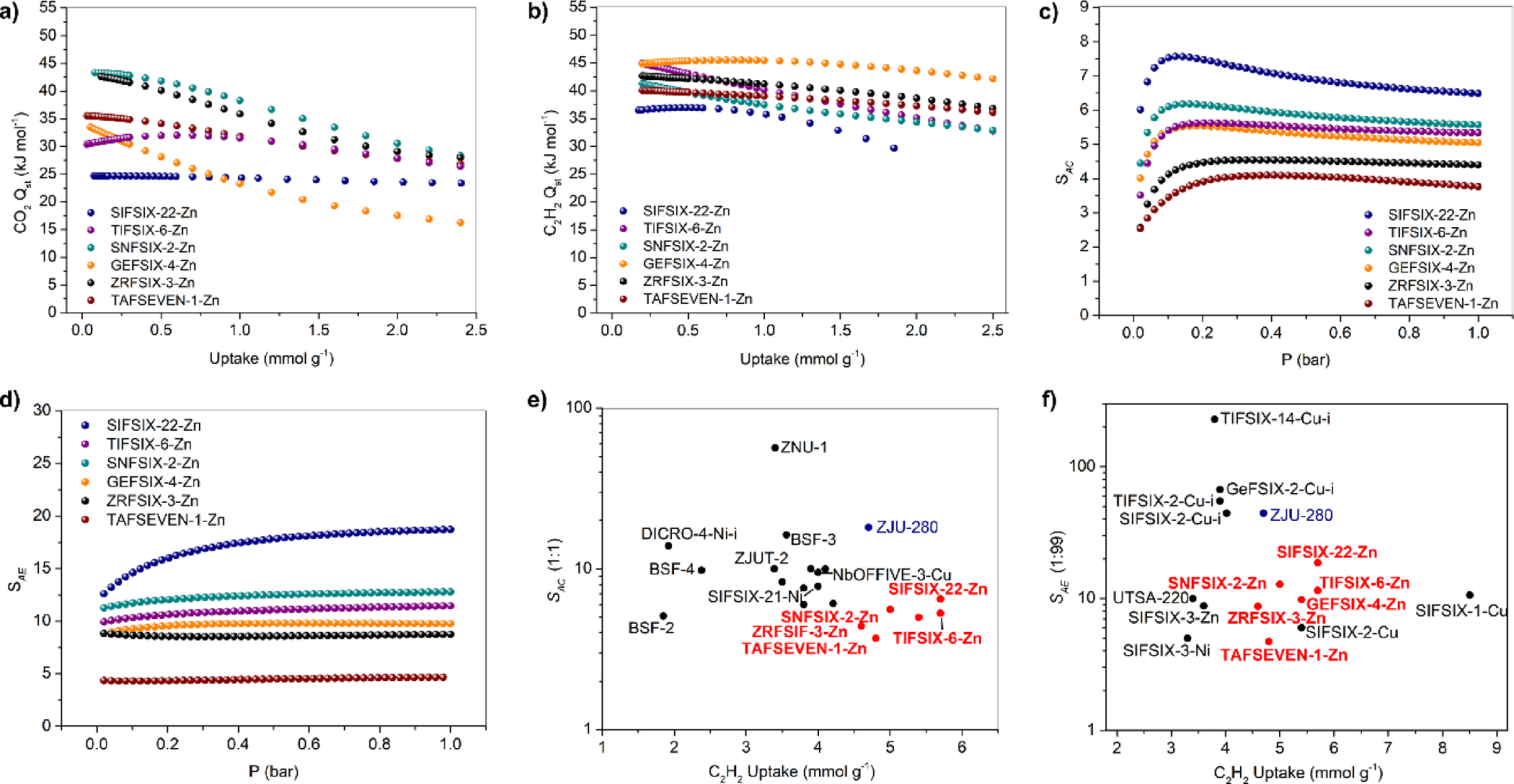
Isosteric heats of adsorption of (a) CO_2_ and (b) C_2_H_2_ on **SIFSIX-22-Zn**, **TIFSIX-6-Zn**, **GEFSIX-4-Zn**, **SNFSIX-2-Zn**, **ZRFSIX-3-Zn**, and **TAFSEVEN-1-Zn**. Ideal adsorbed solution theory (IAST) selectivity determined for (c) 1:1 C_2_H_2_/CO_2_ (*S*_AC_) and (d) 1:99 C_2_H_2_/C_2_H_4_ (*S*_AE_) for **SIFSIX-22-Zn, TIFSIX-6-Zn**, **GEFSIX-4-Zn**, **SNFSIX-2-Zn**, **ZRFSIX-3-Zn**, and **TAFSEVEN-1-Zn**. Comparative plots of leading HCNs with respect to (e) *S*_AC_ and (f) *S*_AE_ versus C_2_H_2_ uptake.

Isosteric heats of sorption (*Q*_st_) were calculated for CO_2_ and C2 gases for all six adsorbents (Figure 5a,b). Low loading *Q*_st_ values for CO_2_ varied from 43.3 and 42.6 kJ mol^–1^ for **SNFSIX-2-Zn** and **ZRFSIX-3-Zn**, respectively, to 30.4 and 24.7 kJ mol^–1^ for **TIFSIX-6-Zn** and **SIFSIX-22-Zn**, respectively. Low loading *Q*_st_ values for C_2_H_2_ were determined to be relatively higher with a narrower range, from 44.8 kJ mol^–1^ for **TIFSIX-6-Zn** and **GEFSIX-4-Zn** to 36.5 kJ mol^–1^ for **SIFSIX-22-Zn**. In contrast, *Q*_st_ values for C_2_H_4_ and C_2_H_6_ range from 33.3 to 31.6 kJ mol^–1^ for C_2_H_4_ and 32.7 to 31.1 kJ mol^–1^ for C_2_H_6_ (Figures S22, S23). Overall, the affinity for C_2_H_2_ was highest, encouraging us to evaluate the selectivity of the six HUMs in the context of C_2_H_2_/CO_2_ and C_2_H_2_/C_2_H_4_ separations.

Calculations conducted using ideal adsorbed solution theory (IAST) indicate that C_2_H_2_/CO_2_ (1:1) selectivities (*S*_AC_) vary between 6.5 (**SIFSIX-22-Zn**) and 3.8 (**TAFSEVEN-1-Zn**) at 1 bar. C_2_H_2_/C_2_H_4_ (1:99) selectivities (*S*_AE_) at 1 bar vary between 18.7 (**SIFSIX-22-Zn**) and 4.7 (**TAFSEVEN-1-Zn**) (Figure 5c,d). Perhaps most notable is that, despite the absence of any apparent correlation between pure component *Q*_st_ values for adsorbents and different gases, *S*_AC_ and *S*_AE_ values follow a clear trend, that is, **SIFSIX-22-Zn** > **SNFSIX-2-Zn** > **TIFSIX-6-Zn** > **GEFSIX-4-Zn** > **ZRFSIX-3-Zn** > **TAFSEVEN-1-Zn**. This correlation suggests that the identity of inorganic anions is responsible for the varying affinity toward acetylene in these HUMs and that the sorption properties are impacted by incorporation of different pillars. No correlation between *S*_AC_ and *S*_AE_ values with the electronegativity of the central atom of the anion was evident, suggesting a need for calculation of pore surface charges and in-depth detailed computational studies on this system to fully elucidate the observed affinities toward C_2_H_2_.

The calculated selectivity values of these adsorbents are moderate but when viewed together with their relatively high uptakes, it is apparent that these **fsc** networks address the trade-off between selectivity and uptake (Figure 5e,f). When compared to other C_2_H_2_ selective sorbents, **SIFSIX-22-Zn** and **TIFSIX-6-Zn** show a rare combination of strong selectivity and high uptake, indicating that further exploration of this platform of materials has the potential to produce adsorbents with strong overall performance.

## Conclusions

We report herein the highly modular **fsc** topology HUM platform which enabled us to explore the effect of changing inorganic pillar on gas sorption properties. The use of SiF_6_^2–^, TiF_6_^2–^, GeF_6_^2–^, SnF_6_^2–^ ZrF_6_^2–^, and TaF_7_^2–^ in combination with Zn^2+^ and the tetratopic **tepb** ligand afforded a family of HUMs: **SIFSIX-22-Zn**, **TIFSIX-6-Zn**, **GEFSIX-4-Zn**, **SNFSIX-2-Zn**, and **ZRFSIX-3-Zn** and the first TaF_7_^**2–**^-pillared HUM, **TAFSEVEN-1-Zn_._** The crystal structures were determined by single-crystal X-ray diffraction and enabled systematic studies of structure–property relationships. Each framework features narrow pore windows, yet significant pore cavities between the zinc(II)-**tepb** layers. This is unusual in HUMs and presents a general strategy that could address the trade-off between uptake and selectivity that is common in adsorbents. Variation of the inorganic pillar resulted in a trend in relative C_2_H_2_ affinity as follows: **SIFSIX-22-Zn** > **SNFSIX-2-Zn** > **TIFSIX-6-Zn** > **GEFSIX-4-Zn** > **ZRFSIX-3-Zn** > **TAFSEVEN-1-Zn**. Overall, this work highlights the modular nature of the **fsc** HUM platform and that substitution of the inorganic pillar impacts structure–property relationships.

## Supporting Information Available

Single-crystal X-ray diffraction data, powder X-ray diffraction patterns, thermal gravimetric analyses, pore analyses, gas sorption isotherms, and IAST calculations for all materials (PDF)

## Abbreviations

SCXRD: single crystal X-ray diffraction
PXRD: powder X-ray diffraction
TGA: thermal gravimetric analysis
tepb: 1,2,4,5-tetra(4-pyridyl)benzene.
IAST: ideal adsorbed solution theory
