# Supplementary material for: Pillar Modularity in fsc Topology Hybrid Ultramicroporous Materials Based upon Tetra(4-pyridyl)benzene
Source: Cryst Growth Des. 2022 Aug 19;22(9):5472–80. doi: 10.1021/acs.cgd.2c00561 (PMC9469729; doi:10.1021/acs.cgd.2c00561)
Supplement: Supplementary file 1 — cg2c00561_si_001.pdf [file cg2c00561_si_001.pdf]

Supporting Information

Pillar modularity in fsc topology hybrid ultramicroporous materials based upon tetra(4-pyridyl)benzene

*Debobroto Sensharma[‡], Benjamin H. Wilson[‡], Naveen Kumar, Daniel J. O'Hearn, Michael J. Zaworotko**

Department of Chemical Sciences, Bernal Institute, University of Limerick,

Limerick V94 T9PX, Republic of Ireland.

Email: xtal@ul.ie

Contents

Characterisation 2

(a) Single-Crystal X-ray Diffraction (SCXRD) 2

CSD Search Details 7

(a) Powder X-ray Diffraction (PXRD) Thermogravimetric Analysis (TGA) 9

(b) Pore properties from SCXRD structures 12

Sorption Studies 15

(a) Low-temperature sorption 15

(a) Isosteric heats of adsorption (Q_{st}) 19

(a) IAST Selectivity Calculations 22

(a) Comparisons of Performance Parameters 24

References 25

Characterisation

(a) Single-Crystal X-ray Diffraction (SCXRD)

Table S1. Single-crystal X-ray data for TIFSIX-6-Zn and GEFSIX-4-Zn.

Identification code	**TIFSIX-6-Zn**	**GEFSIX-4-Zn**
Empirical formula	$C_{26}H_{18}F_6N_4TiZn$	$C_{26}H_{18}F_6GeN_4Zn$
Formula weight	613.71	638.40
Temperature/K	295.0	295.0
Crystal system	orthorhombic	orthorhombic
Space group	*Pmma*	*Cmme*
a/Å	24.9143(18)	15.5555(7)
b/Å	7.9073(6)	24.6790(10)
c/Å	9.4003(7)	9.5067(4)
α/°	90	90
β/°	90	90
γ/°	90	90
Volume/Å^3	1851.9(2)	3649.6(3)
Z	2	4
ρ_{calc}g/cm^3	1.101	1.162
μ/mm^{-1}	3.048	2.248
F(000)	616.0	1272.0
Crystal size/mm^3	0.3 × 0.16 × 0.05	0.15 × 0.1 × 0.1
Radiation	CuKα (λ = 1.54178)	CuKα (λ = 1.54178)
2Θ range for data collection/°	9.408 to 137.066	9.302 to 130.362
Index ranges	-29 ≤ h ≤ 29, -8 ≤ k ≤ 9, -11 ≤ l ≤ 10	-18 ≤ h ≤ 17, -29 ≤ k ≤ 28, -11 ≤ l ≤ 11
Reflections collected	18175	17861
Independent reflections	1873 [R_{int} = 0.0461, R_{sigma} = 0.0256]	1673 [R_{int} = 0.0482, R_{sigma} = 0.0248]
Data/restraints/parameters	1873/36/166	1673/0/94
Goodness-of-fit on F^2	1.131	1.076
Final R indexes [I>=2σ (I)]	R_1 = 0.0313, wR_2 = 0.1091	R_1 = 0.0407, wR_2 = 0.1214
Final R indexes [all data]	R_1 = 0.0414, wR_2 = 0.1251	R_1 = 0.0442, wR_2 = 0.1242
Largest diff. peak/hole / e Å^{-3}	0.28/-0.34	0.43/-0.94
CCDC deposition number	2151313	2151312

Table S2. Single-crystal X-ray data for SNFSIX-2-Zn and ZRFSIX-3-Zn.

	**SNFSIX-2-Zn**	**ZRFSIX-3-Zn**
Empirical formula	$C_{26}H_{18}F_6N_4SnZn$	$C_{26}H_{18}F_6N_4ZnZr$
Formula weight	684.50	657.03
Temperature/K	295.0	298.0
Crystal system	orthorhombic	orthorhombic
Space group	*Cmme*	*Pmmm*
a/Å	16.1892(4)	8.1775(4)
b/Å	24.6766(6)	9.5451(4)
c/Å	9.5261(2)	12.3540(5)
α/°	90	90
β/°	90	90
γ/°	90	90
Volume/Å^3	3805.62(15)	964.29(7)
Z	4	1
ρ_{calc}g/cm^3	1.195	1.131
μ/mm^{-1}	6.403	3.383
F(000)	1344.0	326.0
Crystal size/mm^3	0.06 × 0.06 × 0.06	0.05 × 0.05 × 0.05
Radiation	CuKα (λ = 1.54178)	CuKα (λ = 1.54178)
2Θ range for data collection/°	9.284 to 136.682	7.156 to 129.664
Index ranges	-19 ≤ h ≤ 19, -29 ≤ k ≤ 28, -11 ≤ l ≤ 10	-8 ≤ h ≤ 9, -11 ≤ k ≤ 10, -14 ≤ l ≤ 14
Reflections collected	20616	7231
Independent reflections	1866 [R_{int} = 0.0452, R_{sigma} = 0.0239]	986 [R_{int} = 0.0711, R_{sigma} = 0.0349]
Data/restraints/parameters	1866/0/106	986/0/83
Goodness-of-fit on F^2	1.146	1.081
Final R indexes [I>=2σ (I)]	R_1 = 0.0284, wR_2 = 0.0839	R_1 = 0.0283, wR_2 = 0.0730
Final R indexes [all data]	R_1 = 0.0311, wR_2 = 0.0857	R_1 = 0.0353, wR_2 = 0.0776
Largest diff. peak/hole / e Å^{-3}	0.54/-0.22	0.25/-0.38
CCDC deposition number	2151311	2151309

Table S3. Single-crystal X-ray data for TAFSEVEN-1-Zn.

	**TAFSEVEN-1-Zn**
Empirical formula	$C_{26}H_{18}F_7N_4TaZn$
Formula weight	765.76
Temperature/K	298.0
Crystal system	orthorhombic
Space group	*Cmme*
a/Å	15.8313(6)
b/Å	24.8247(10)
c/Å	9.5493(4)
α/°	90
β/°	90
γ/°	90
Volume/Å^3	3752.9(3)
Z	4
ρ_{calc}g/cm^3	1.355
μ/mm^{-1}	6.535
F(000)	1472.0
Crystal size/mm^3	0.08 × 0.08 × 0.02
Radiation	CuKα (λ = 1.54178)
2Θ range for data collection/°	9.26 to 133.346
Index ranges	-18 ≤ h ≤ 18, -29 ≤ k ≤ 24, -11 ≤ l ≤ 11
Reflections collected	22540
Independent reflections	1761 [R_{int} = 0.0450, R_{sigma} = 0.0212]
Data/restraints/parameters	1761/98/148
Goodness-of-fit on F^2	1.084
Final R indexes [I>=2σ (I)]	R_1 = 0.0268, wR_2 = 0.0848
Final R indexes [all data]	R_1 = 0.0301, wR_2 = 0.0885
Largest diff. peak/hole / e Å^{-3}	0.54/-0.81
CCDC deposition number	2151310

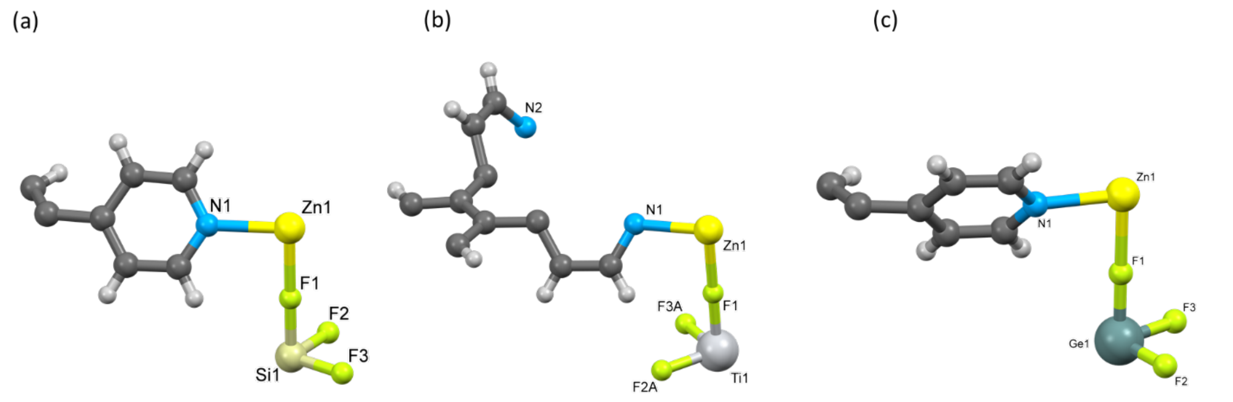

Figure S1. Asymmetric unit for SIFSIX-22-Zn (a), TIFSIX-6-Zn (b) and GEFSIX-4-Zn (c) with non-hydrogen and carbon atoms labelled. Disorder of pyridyl rings of TIFSIX-6-Zn and fluorine atoms of TIFSIX-6-Zn and SNFSIX-2-Zn omitted for clarity. Grey = C, blue = N, green = F.

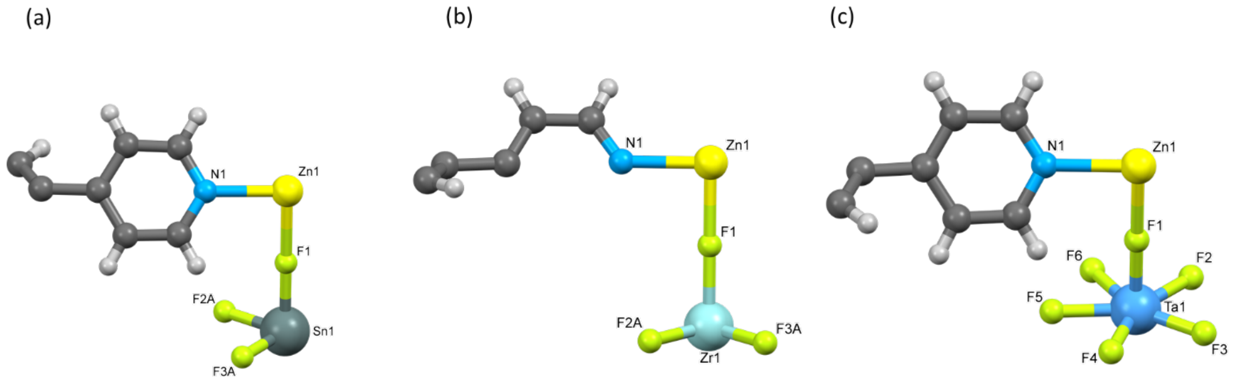

Figure S2. Asymmetric unit for SNFSIX-2-Zn (a), ZRFSIX-3-Zn (b) and TAFSEVEN-1-Zn (c) with non-hydrogen and carbon atoms labelled. Disorder of pyridyl rings and fluorine atoms of ZRFSIX-3-Zn omitted for clarity. Grey = C, blue = N, green = F.

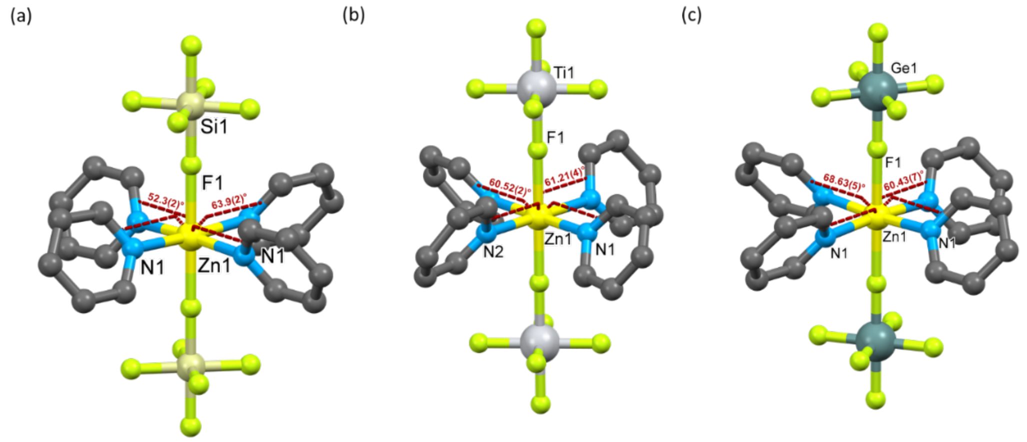

Figure S3. The octahedral coordination environment of zinc in SIFSIX-22-Zn (a), TIFSIX-6-Zn (b) and GEFSIX-4-Zn (c). The angles shown are the torsions between the coordinated pyridyl rings opposite each other. The rest of the ligand and hydrogen atoms are omitted for clarity. Grey = C, blue = N, green = F.

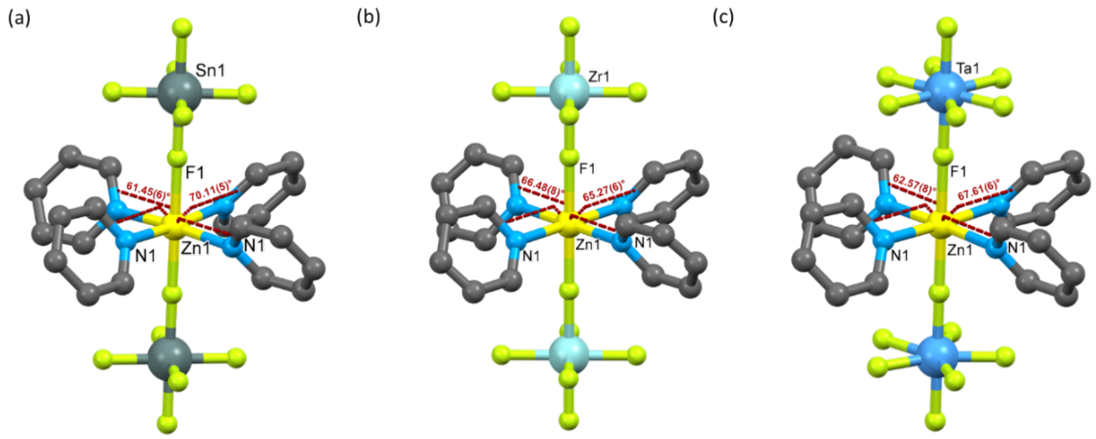

Figure S4. The octahedral coordination environment of zinc in SNFSIX-2-Zn (a), ZRFSIX-3-Zn (b) and TAFSEVEN-1-Zn (c). The angles shown are the torsions between the coordinated pyridyl rings opposite each other. The rest of the ligand and hydrogen atoms are omitted for clarity. Grey = C, blue = N, green = F.

CSD Search Details

With the search queries shown below, the CSD was searched with results restricted to R1 <= 0.075. 20 structures were found, and the statistical analysis was performed in Mercury.

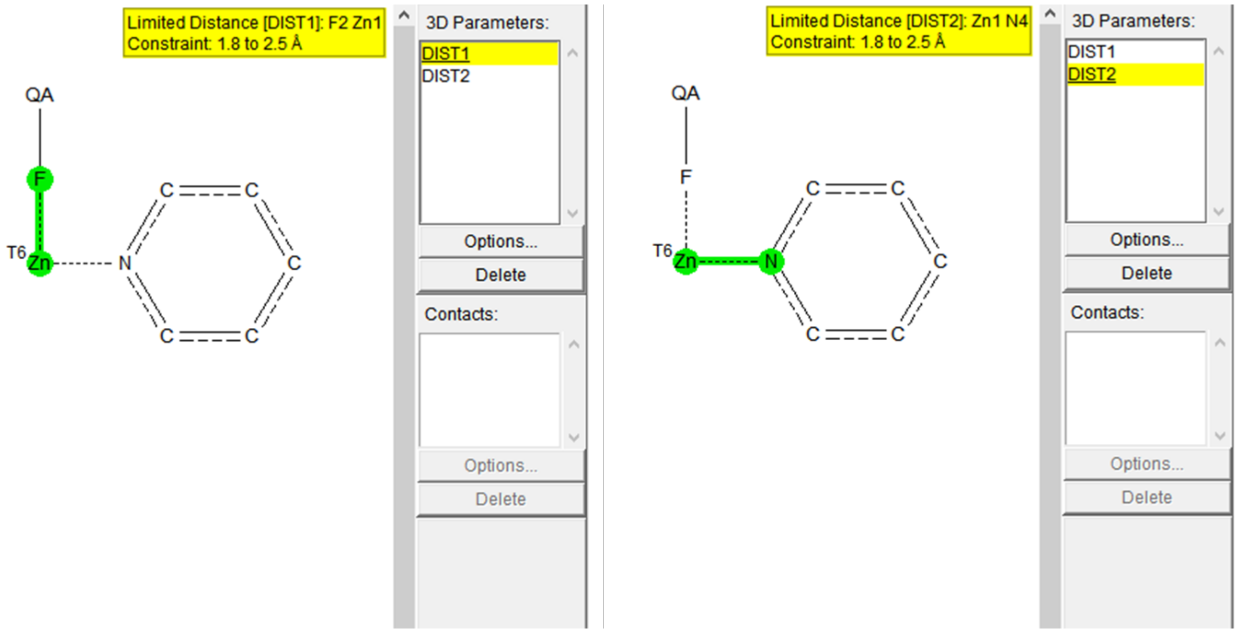

Figure S5. CSD search queries for Zn-F (left) and N-Zn (right) distances. QA = Si, Ti, Ge, Sn, Zr and Ta.

With the search queries shown below, the CSD was searched with 0 structures found.

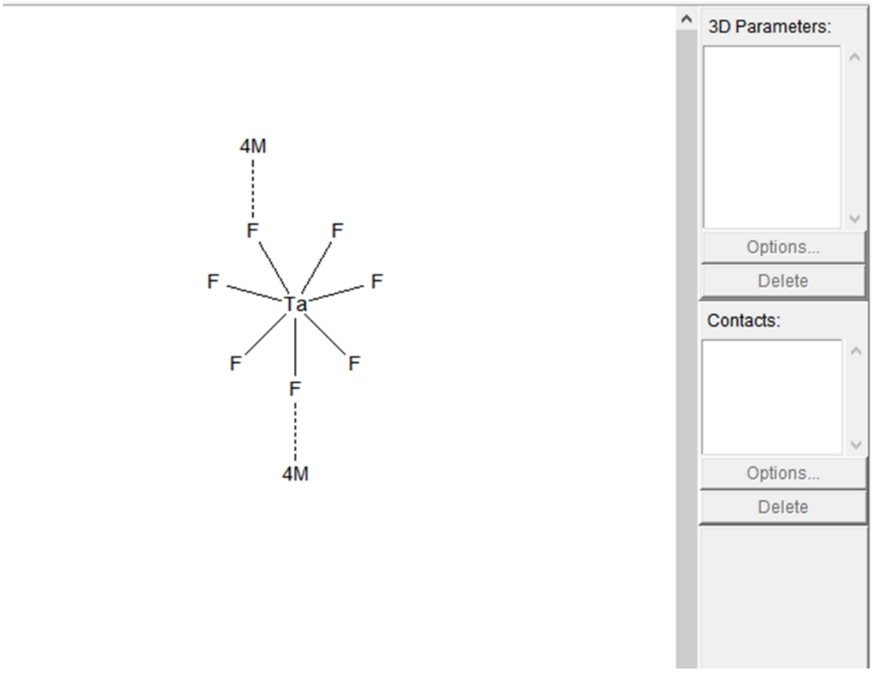

Figure S6. CSD search queries TaF_7^{2-} bridging two metal centres distances. 4M = any metal.

Table S4. Axial Zn-F and equatorial Zn-N bond lengths.

	Zn-F bond Distance (Å)	Zn-N bond Distance (Å)
**SIFSIX-22-Zn**	2.064(2)	2.151(1)
**TIFSIX-6-Zn**	2.0655(14)	2.147(2), 2.149(2)
**GEFSIX-4-Zn**	2.090(2)	2.137(2)
**SNFSIX-2-Zn**	2.0680(18)	2.143(2)
**ZRFSIX-3-Zn**	2.066(3)	2.147(3)
**TAFSEVEN-1-Zn**	2.040(2)	2.176(3)

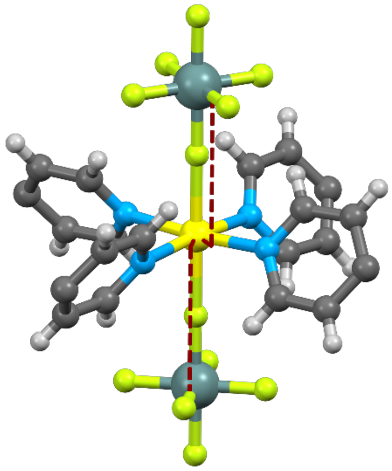

Figure S7. Representation of F-M-M-F torsion used for calculation of pillar rotation angle. Grey = C, blue = N, green = F, yellow = Zn, grey = M.

Table S5. MF_n^{2-} pillar rotation angle (F-M-M-F torsion).

	MF_n^{2-} pillar rotation angle (°)
**SIFSIX-22-Zn**	20.093(2)
**TIFSIX-6-Zn**	0
**GEFSIX-4-Zn**	55.0(2)
**SNFSIX-2-Zn**	4.9(5), 23.0(5)
**ZRFSIX-3-Zn**	0
**TAFSEVEN-1-Zn**	9.8(8)

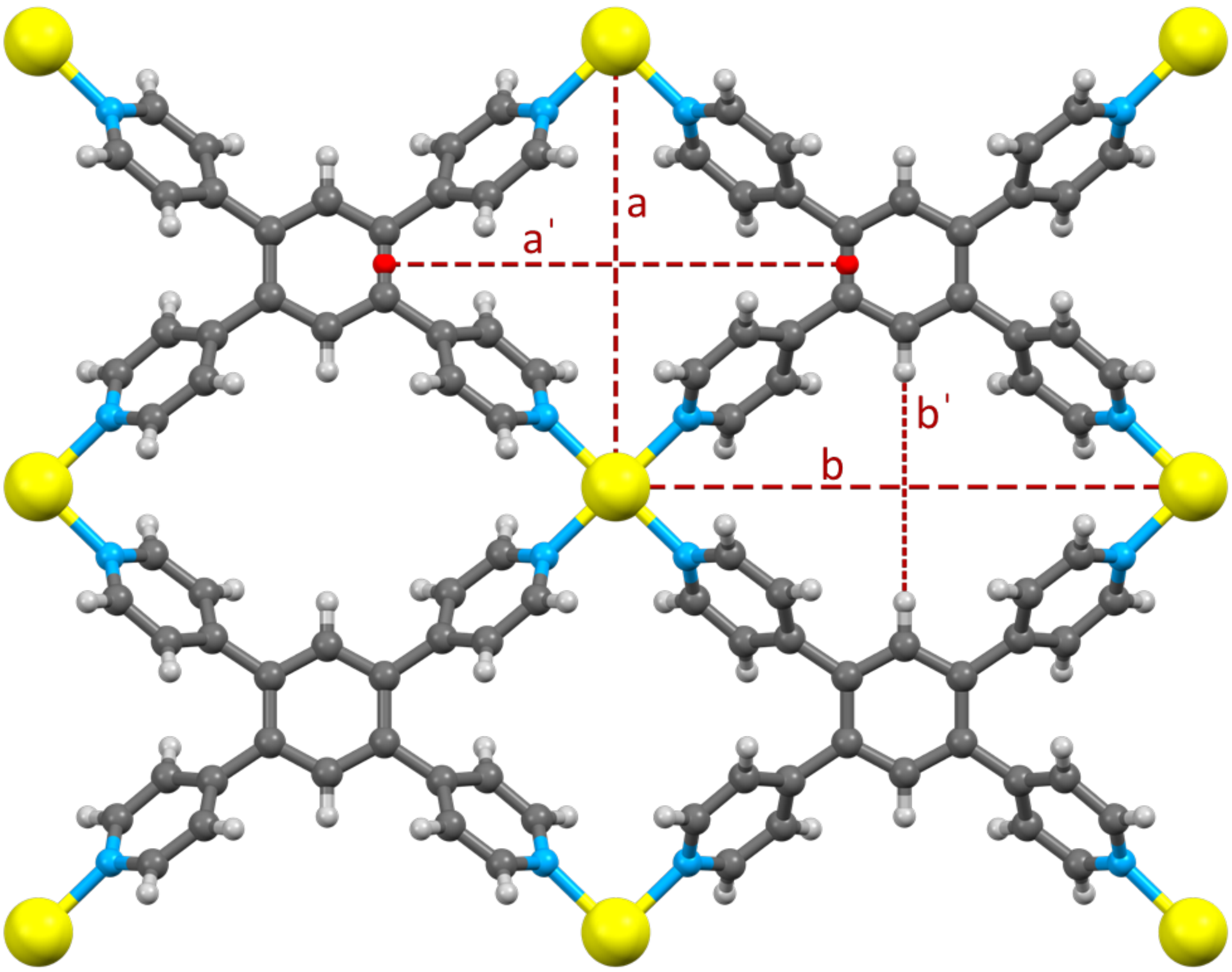

Figure S8. Two windows exist in the Zn-tepb 2-dimensional layer. Window A with dimensions a x a' and window B with dimensions b x b'.

Table S6. Window dimensions.

	Window A: a x a' (Å)	Window B: b x b' (Å)
**SIFSIX-22-Zn**	9.439(1) x 9.988(3)	12.4279(7) x 4.8948(5)
**TIFSIX-6-Zn**	9.4003(7) x 10.015(3)	12.4572(9) x 4.8206(2)
**GEFSIX-4-Zn**	9.5067(4) x 9.893(5)	12.3395(5) x 4.9682(2)
**SNFSIX-2-Zn**	9.5261(2) x 9.895(4)	12.3383(3) x 49903(1)
**ZRFSIX-3-Zn**	9.5451(4) x 9.908(2)	12.3540(5) x 4.9695(2)
**TAFSEVEN-1-Zn**	9.5493(4) x 9.64(5)	12.4124(5) x 4.9859(2)

(a) Powder X-ray Diffraction (PXRD) Thermogravimetric Analysis (TGA)

Powder X-ray diffraction (PXRD) and thermogravimetric analysis (TGA) was performed on as-synthesised and activated **SIFSIX-22-Zn**, **TIFSIX-6-Zn**, **GEFSIX-4-Zn**, **SNFSIX-2-Zn**, **ZRFSIX-3-Zn** and **TAFSEVEN-1-Zn**. For stability testing, PXRD patterns were recorded of activated samples of **SIFSIX-22-Zn**, **TIFSIX-6-Zn**, **GEFSIX-4-Zn**, **SNFSIX-2-Zn**, **ZRFSIX-3-Zn** and **TAFSEVEN-1-Zn** soaked in methanol at room temperature after one week, as well placed in a humidity chamber at 40 °C and 75% relative humidity after 24 hours and one week.

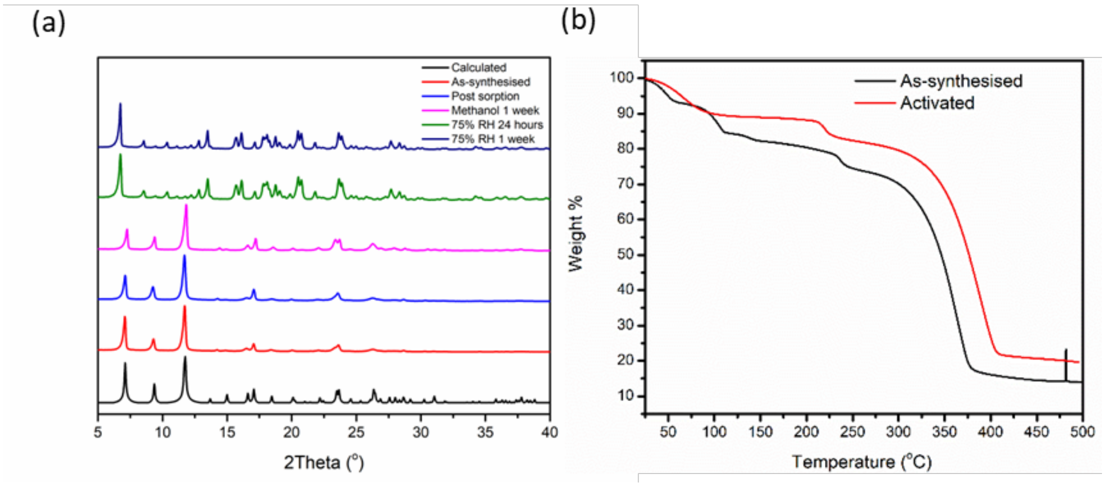

Figure S9. PXRD patterns (left) and thermogravimetric traces (right) of SIFSIX-22-Zn.

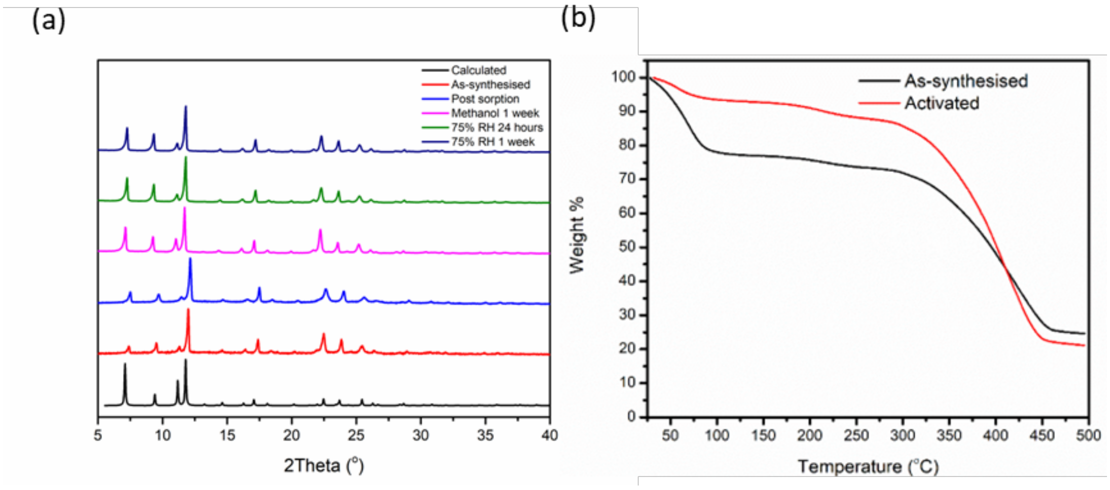

Figure S10. PXRD patterns (left) and thermogravimetric traces (right) of TIFSIX-6-Zn.

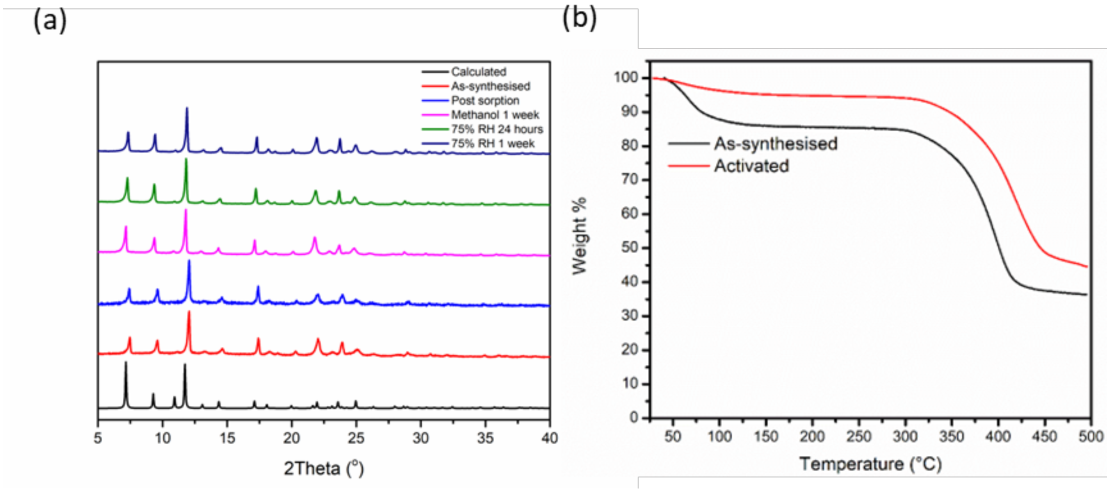

Figure S11. PXRD patterns (left) and thermogravimetric traces (right) of SNFSIX-2-Zn.

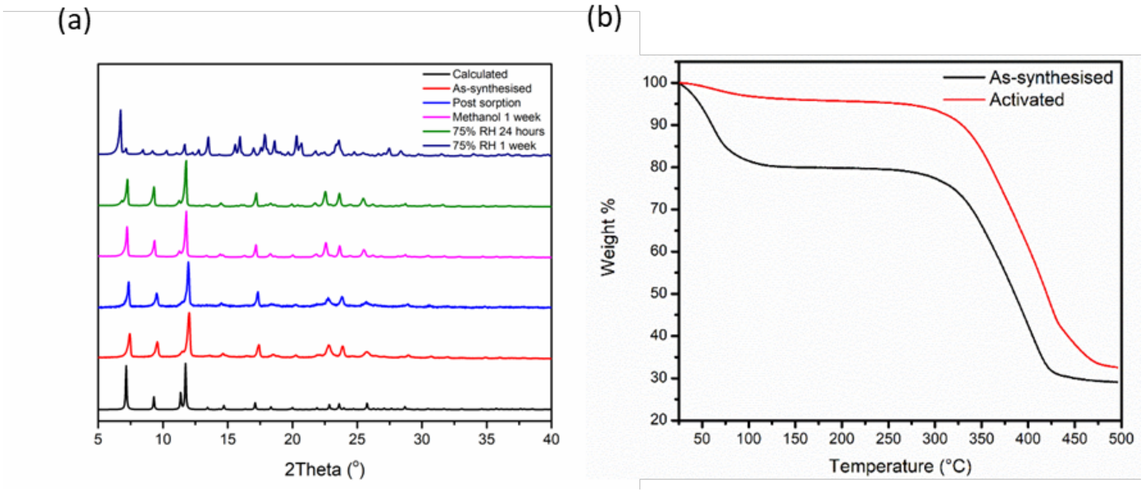

Figure S12. PXRD patterns (left) and thermogravimetric traces (right) of GEFSIX-4-Zn.

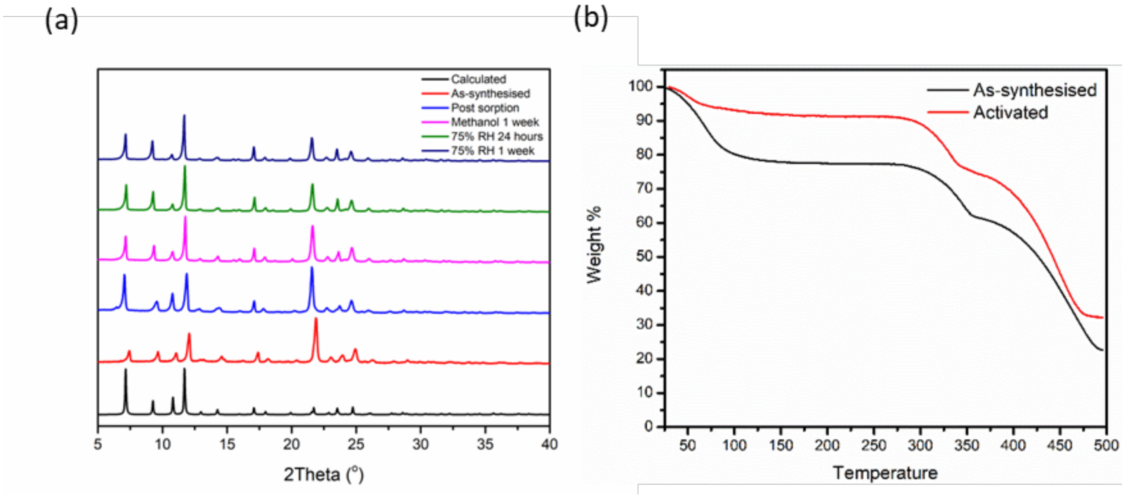

Figure S13. PXRD patterns (left) and thermogravimetric traces (right) of ZRFSIX-3-Zn.

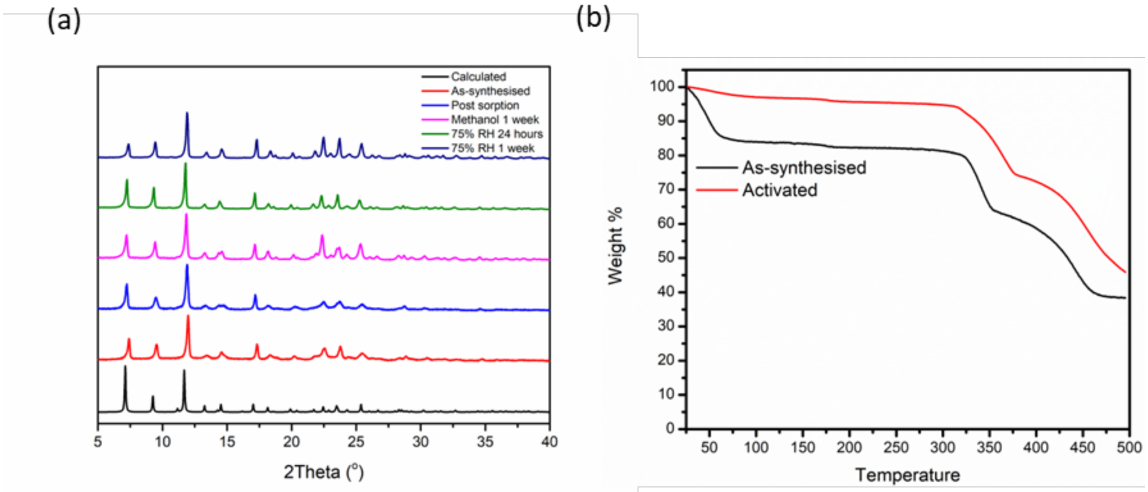

Figure S14. PXRD patterns (left) and thermogravimetric traces (right) of TAFSEVEN-1-Zn.

(b) Pore properties from SCXRD structures

The void volume per formula unit, limiting pore diameter and max pore diameter were calculated using Poreblazer v4.0,[1] omitting any disorder of the ligand and/or inorganic pillar.

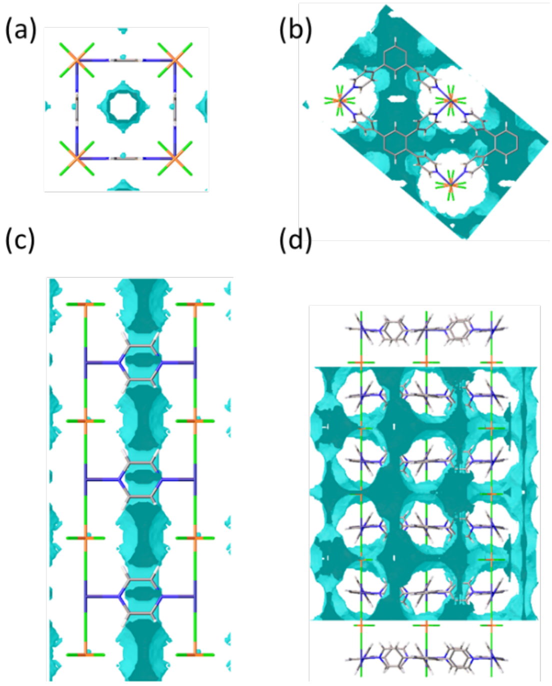

Figure S15. The pores present in SIFSIX-3-Zn (a and c) compared to the pores present in SIFSIX-22-Zn (b and d).

Table S7. Crystallographic pore parameters for SIFSIX-22-Zn, TIFSIX-6-Zn, GEFSIX-4-Zn, SNFSIX-2-Zn, ZRFSIX-3-Zn and TAFSEVEN-1-Zn.

Compound	Interlayer M-M distance (Å)	Total volume per formula unit (Å^3)	Crystallographic void volume per formula unit (Å^3)	Void volume percent	Limiting pore diameter (Å)	Max. pore diameter (Å)
**SIFSIX-22-Zn**	7.568	887.8	111.14	12.5%	3.18	5.84
**GEFSIX-4-Zn**	7.778	912.4	125.62	13.8%	3.23	5.92
**TIFSIX-6-Zn**	7.907	925.9	99.21	10.7%	3.12	4.71
**TAFSEVEN-1-Zn**	7.916	938.2	90.49	9.6%	3.31	4.84
**SNFSIX-2-Zn**	8.095	951.4	133.40	11.9%	3.39	5.78
**ZRFSIX-3-Zn**	8.177	964.3	135.20	14.2%	3.71	4.95

The pore limiting diameter and maximum pore diameter in **SIFSIX-22-Zn** and several representative hybrid coordination networks (HCNs) was determined from their crystal structures. Effective channel

diameters were calculated using the "calcvoid" function in Olex2. Van der Waals radii were used as obtained from the CCDC.[1, 2] The calculation was done to a surface resolution of 0.1 Å in the vectorised mode. Atoms associated with guest molecules or coordinated solvent were manually removed before the calculations.

Table S8. Crystallographically determined pore dimensions in representative HCNs.

Compound	CSD Refcode	Pore Limiting Diameter (Å)	Diameter of the Largest Spherical Void (Å)	Remarks	Ref.
**SIFSIX-1-Zn**	ZESFUY	8	9.6		4
**SIFSIX-1-Cu**	GORWUF	7.2	8		5
**TIFSIX-1-Cu**	PETWIW	7.2	8.8		6
**SNFSIX-1-Cu**	PETWES	7.2	8.8		6
**SIFSIX-2-Zn**	WONZOP	10	10.8		7
**SIFSIX-2-Cu**	YEMTER	9.6	10.8	Solvent removed	8
**SIFSIX-2-Cu-i**	YEMTIV	3.2	3.6		8
**SIFSIX-3-Zn**	FUDQIF	3.2	4		9
**SIFSIX-3-Cu**	WONKOB	3.2	4		10
**SIFSIX-4-Zn**	WONZUV	9.2	11.2		7
**SIFSIX-5-Zn-i**	LIFWII	5.2	6.4	Solvent removed	11
**SIFSIX-6-Zn-i**	LIFWOO	4.4	5.2	Solvent removed	11
**SIFSIX-7-Cu**	HAPKUG	9.6	10		12
**SIFSIX-8-Cu**	GIKPIB	7.6	8.4		13
**SIFSIX-13-Zn**	FORKOO	10.8	12.8	Disordered	14
**SIFSIX-14-Cu-i**	WIBWEM	3.2	3.6		15
**SIFSIX-15-Zn-i**	NATHIC	10.4	10.8		16
**SIFSIX-15-Cu-i**	NATKAX	10	10.4		16
**SIFSIX-16-Zn-i**	NATHOI	8	8.4		16
**SIFSIX-18-Cd**	KIKDOZ	4	5.6		17
**SIFSIX-19-Cu-i**	DOCVEZ	3.6	4.4	Solvent removed	18
**TIFSIX-19-Cu-i**	DOCVAW	3.2	4.4	Solvent removed	18
**MOOFOUR-1-Co**	YEZKOF	2.8	6.4	Solvent removed	19
**MOOFOUR-1-Ni**	YEZKUL	2.8	6	Solvent removed	19
**NbOFFIVE-1-Cu**	CEHRIS	2.4	3.6		20
**NbOFFIVE-1-Ni**	ARAHIM	2.8	3.6		21
**AlFFIVE-1-Ni**	DAXNEY	2.8	3.6	Disordered	22
**DICRO-2-Ni-i**	PUSJUK	2.8	3.6		23
**DICRO-5-Co-i**	TAQGOK	4.4	5.2		24
**DICRO-6-Co-i**	TAQGUQ	2.4	4.4		24
**Tripp-Cu-SIFSIX**	WAHNOL	7.2	16		25

**fsc-2-SIFSIX**	ZAHLAY	7.4	9.2	Coordinated waters on paddlewheel removed	26
**CPM-131**	CECGOJ	4.4	6.4	Coordinated waters on porphyrinic Fe removed	27
**SIFSIX-22-Zn**	this work	3.6	6		this work
**TIFSIX-6-Zn**	this work	3.6	5.2		this work
**GEFSIX-4-Zn**	this work	3.6	6.0		this work
**SNFSIX-2-Zn**	this work	3.6	5.6		this work
**ZRFSIX-3-Zn**	this work	4.0	5.2		this work
**TAFSEVEN-1-Zn**	this work	3.6	5.2		this work

Sorption Studies

(a) Low-temperature sorption

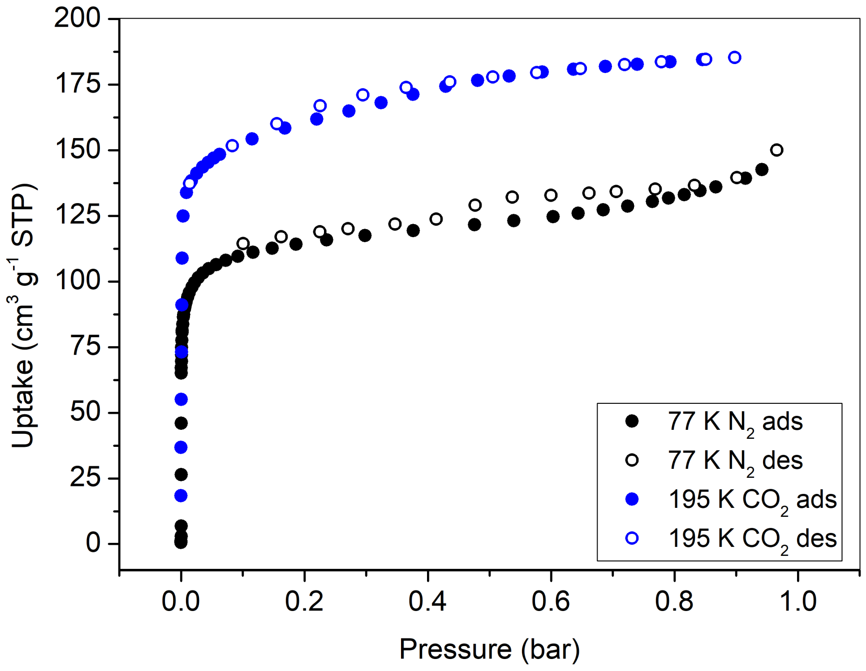

Figure S16. 77 K N_2 (left) and 195 K CO_2 (right) isotherms for SIFSIX-22-Zn.

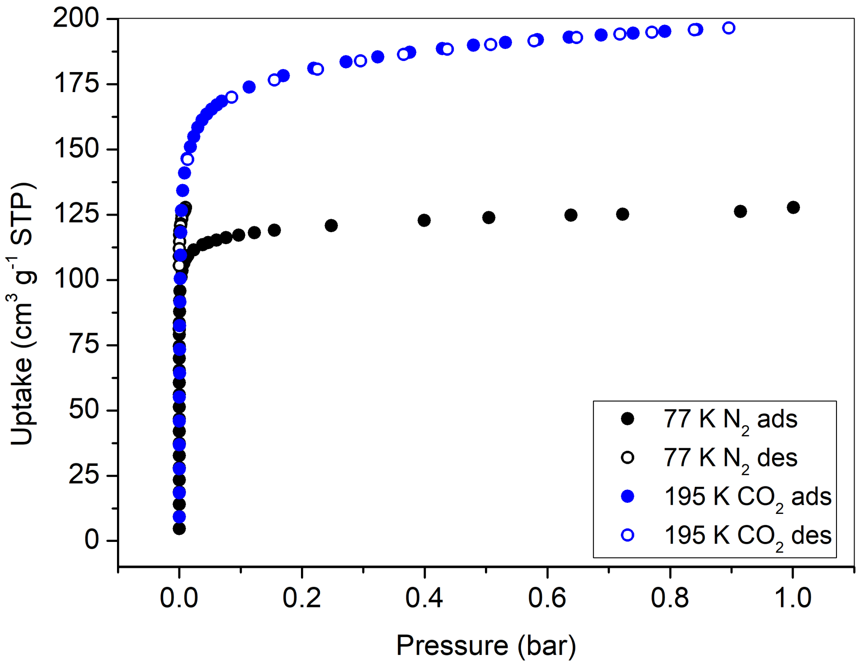

Figure S17. 77 K N_2 (left) and 195 K CO_2 (right) isotherms for TIFSIX-6-Zn.

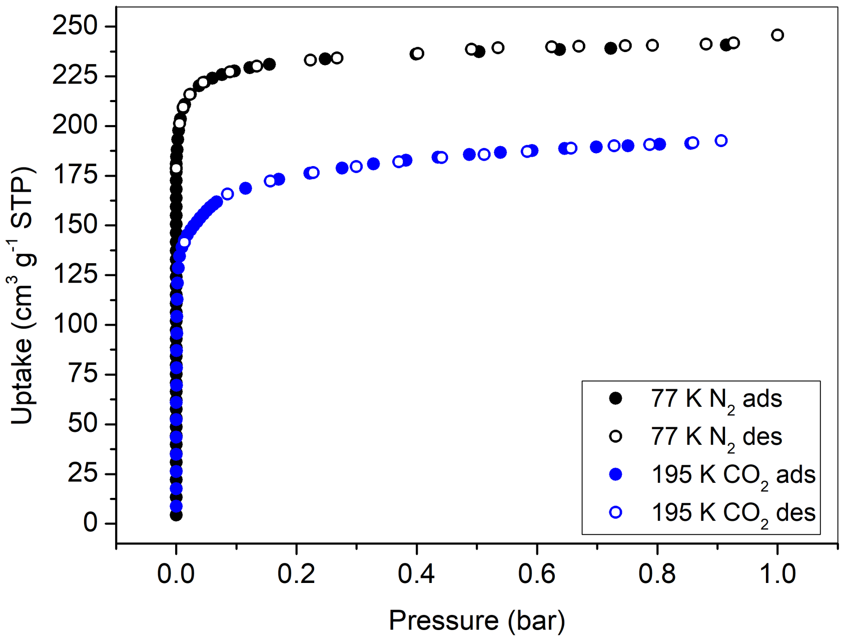

Figure S18. 77 K N_2 (left) and 195 K CO_2 (right) isotherms for GEFSIX-4-Zn.

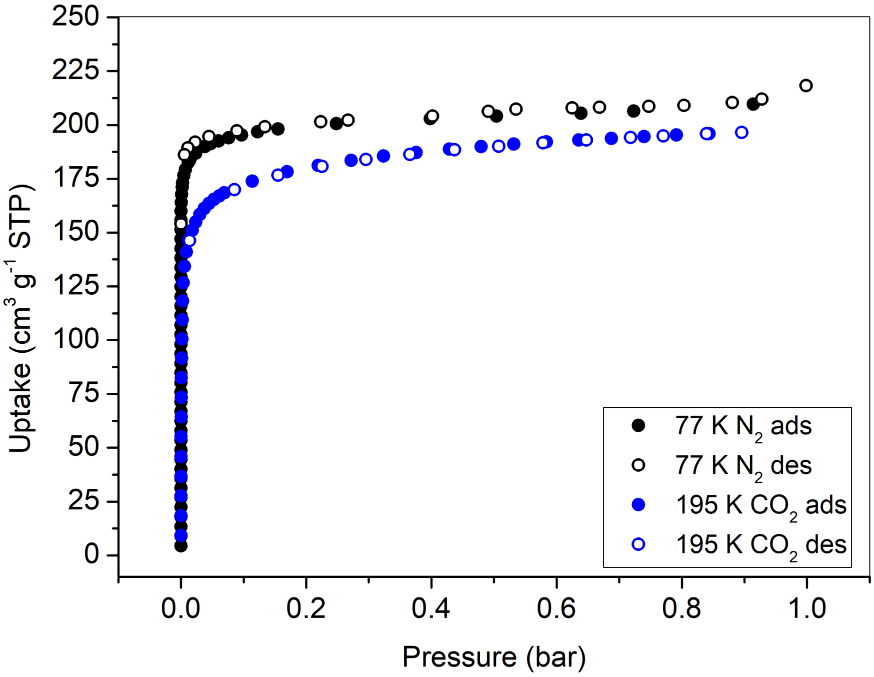

Figure S19. 77 K N_2 (left) and 195 K CO_2 (right) isotherms for SNFSIX-2-Zn.

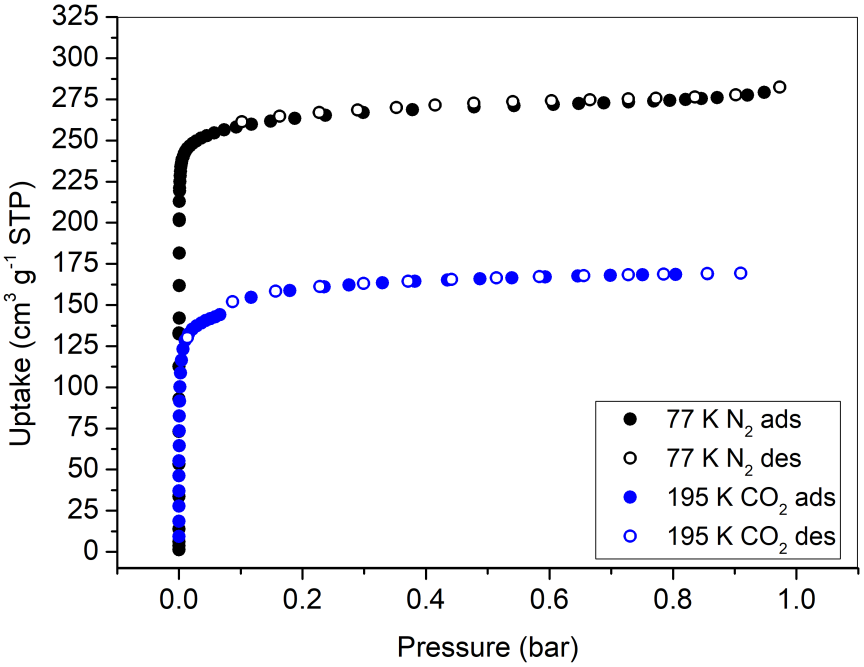

Figure S20. 77 K N_2 (left) and 195 K CO_2 (right) isotherms for ZRFSIX-3-Zn.

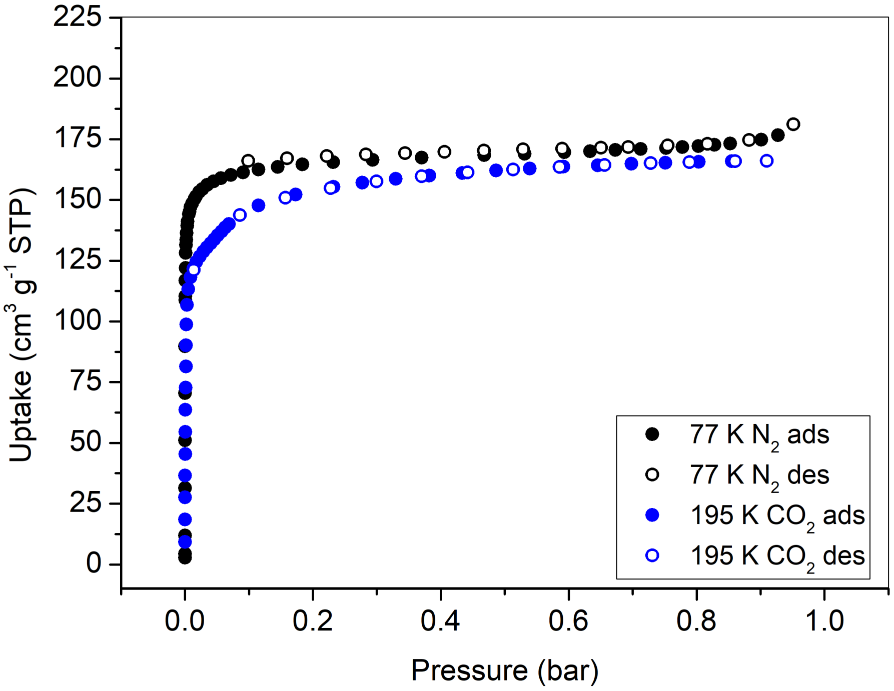

Figure S21. 77 K N_2 (left) and 195 K CO_2 (right) isotherms for TAFSEVEN-1-Zn.

Table S9. Summary of BET surface areas derived from 77 K N_2 and 195 K CO_2 isotherms.

SIFSIX-22-Zn	**N_2 77 K**	**CO_2 195 K**
**BET surface area**	414.2656 ± 1.9745 $m^2 g^{-1}$	387.2491 ± 0.4859 $m^2 g^{-1}$
**Slope**	0.23541 ± 0.00112 g $mmol^{-1}$	0.26388 ± 0.00033 g $mmol^{-1}$
**Y-intercept**	0.00009 ± 0.00001 g $mmol^{-1}$	0.00049 ± 0.00002 g $mmol^{-1}$
**C**	2,631.380519	544.871743
**Q_m**	4.24630 mmol g^{-1}	3.78258 mmol g^{-1}
**Correlation coefficient**	0.9999432	0.9999968
**Molecular cross-sectional area:**	0.1620 nm^2	0.1700 nm^2
TIFSIX-6-Zn	**N_2 77 K**	**CO_2 195 K**
**BET surface area**	478.1271 ± 0.6246 m^2/g	396.8358 ± 1.2875 m^2/g
**Slope**	0.20399 ± 0.00027 g/mmol	0.25750 ± 0.00084 g/mmol
**Y-intercept**	0.00006 ± 0.00001 g/mmol	0.00048 ± 0.00003 g/mmol
**C**	3,665.653362	533.333537
**Q_m**	4.90090 mmol/g	3.87623 mmol/g
**Correlation coefficient**	0.9999949	0.9999525
**Molecular cross-sectional area:**	0.1620 nm^2	0.1700 nm^2
GEFSIX-4-Zn	**N_2 77 K**	**CO_2 195 K**
**BET surface area**	925.1362 ± 2.9378 m^2/g	700.0410 ± 2.4580 m^2/g
**Slope**	0.10542 ± 0.00033 g/mmol	0.14606 ± 0.00051 g/mmol
**Y-intercept**	0.00003 ± 0.00001 g/mmol	0.00019 ± 0.00002 g/mmol
**C**	3,520.031131	777.217147
**Q_m**	9.48283 mmol/g	6.83788 mmol/g
**Correlation coefficient**	0.9999748	0.9999321
**Molecular cross-sectional area:**	0.1620 nm^2	0.1700 nm^2
SNFSIX-2-Zn	**N_2 77 K**	**CO_2 195 K**
**BET surface area**	798.0751 ± 1.1745 m^2/g	615.4063 ± 0.9861 m^2/g
**Slope**	0.12222 ± 0.00018 g/mmol	0.16618 ± 0.00027 g/mmol
**Y-intercept**	0.00002 ± 0.00000 g/mmol	0.00017 ± 0.00001 g/mmol
**C**	5,725.873086	956.734384
**Q_m**	8.18043 mmol/g	6.01119 mmol/g
**Correlation coefficient**	0.9999946	0.9999884
**Molecular cross-sectional area:**	0.1620 nm^2	0.1700 nm^2
ZRFSIX-3-Zn	**N_2 77 K**	**CO_2 195 K**
**BET surface area**	1,062.0413 ± 0.8396 m^2/g	625.8791 ± 0.5327 m^2/g
**Slope**	0.004098 ± 0.000003 g/cm^3 STP	0.16345 ± 0.00014 g/mmol
**Y-intercept**	0.000001 ± 0.000000 g/cm^3 STP	0.00013 ± 0.00000 g/mmol
**C**	6,628.078918	1,295.174176
**Q_m**	244.0028 cm^3/g STP	6.11348 mmol/g
**Correlation coefficient**	0.9999978	0.9999985
**Molecular cross-sectional area:**	0.1620 nm^2	0.1700 nm^2
TAFSEVEN-1-Zn	**N_2 77 K**	**CO_2 195 K**
**BET surface area**	652.8849 ± 1.6835 m^2/g	607.4565 ± 1.4934 m^2/g
**Slope**	0.006665 ± 0.000017 g/cm^3 STP	0.16824 ± 0.00041 g/mmol
**Y-intercept**	0.000002 ± 0.000000 g/cm^3 STP	0.00029 ± 0.00002 g/mmol
**C**	3,645.074690	576.297772
**Q_m**	149.9996 cm^3/g STP	5.93353 mmol/g
**Correlation coefficient**	0.9999767	0.9999728
**Molecular cross-sectional area:**	0.1620 nm^2	0.1700 nm^2

(a) Isosteric heats of adsorption (Q_{st})

Isosteric heats of adsorption (Q_{st}) values were determined using the adsorption branches of isotherms of each adsorbate at 273 K and 298 K. A Virial equation of state (Equation 1) was used to fit the isotherm data of C_2H_2, C_2H_4, C_2H_6 and CO_2 on **SIFSIX-22-Zn**, **TIFSIX-6-Zn**, **GEFSIX-4-Zn**, **SNFSIX-2-Zn**, **ZRFSIX-3-Zn** and **TAFSEVEN-1-Zn**. P stands for the pressure in Pa, *N* is the adsorbed amount in mmol g^{-1}, T is the temperature in K, a_i and b_i are virial coefficients, and *m* and *n* are the number of coefficients used to describe the isotherms. Q_{st} is the coverage-dependent enthalpy of adsorption and R is the universal gas constant. All fitting was performed using Origin Pro 2016.

$$\ln P = \ln N + \sum_{i=0}^{m} a_i N_i + \sum_{i=0}^{n} \binom{n}{k} b_i N_i \qquad \textbf{Eqn. 1}$$

Q_{st} was calculated from the virial model using the equation below.

$$-Q_{st} = -R \sum_{i=0}^{m} a_i N_i \qquad \textbf{Eqn. 2}$$

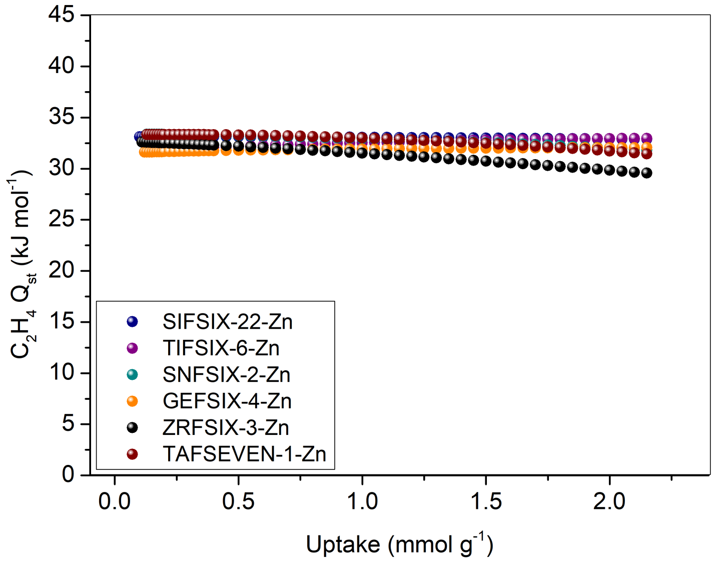

Figure S22. Isosteric heats of adsorption of C_2H_4 on SIFSIX-22-Zn, TIFSIX-6-Zn, GEFSIX-4-Zn, SNFSIX-2-Zn, ZRFSIX-3-Zn and TAFSEVEN-1-Zn plotted as a function of loading.

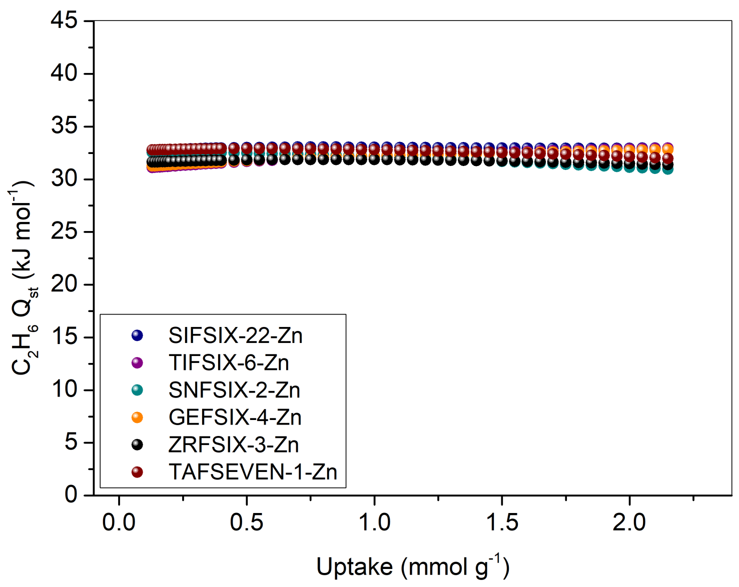

Figure S23. Isosteric heats of adsorption of C_2H_6 on SIFSIX-22-Zn, TIFSIX-6-Zn, GEFSIX-4-Zn, SNFSIX-2-Zn, ZRFSIX-3-Zn and TAFSEVEN-1-Zn plotted as a function of loading.

Virial fit parameters are presented in Tables S10-S13.

Table S10. Virial fit parameters for CO_2 sorption on SIFSIX-22-Zn, TIFSIX-6-Zn, GEFSIX-4-Zn, SNFSIX-2-Zn, ZRFSIX-3-Zn and **TAFSEVEN-1-Zn.**

	**SIFSIX-22-Zn**	**TIFSIX-6-Zn**	**GEFSIX-4-Zn**	**SNFSIX-2-Zn**	**ZRFSIX-3-Zn**	**TAFSEVEN-1-Zn**
**Temperatures (K)**	273, 298	273, 298	273, 298	273, 298	273, 298	273, 298
**Adj. R^2**	0.99977	0.99973	0.99979	0.99977	0.99979	0.99991
**a_0**	-2972.31	-3633.9036	-4103.89	-5217.19	-5185.23	-4283.01
**a_1**	-12.3598	-806.27739	1555.509	-15.6753	392.6569	171.0314
**a_2**	82.50563	886.22009	-157.524	947.9344	878.6786	473.8865
**a_3**	-25.8824	-271.55625	-135.917	-377.217	-484.877	-236.399
**a_4**	2.21796	37.69458	44.47035	58.26887	93.42663	42.80281
**a_5**	0	-2.58231	-3.47212	-4.18338	-7.23737	-3.02648
**b_0**	12.85476	18.88064	21.01995	24.56661	24.0118	22.19623
**b_1**	0.46529	3.53634	-5.06336	0.34046	-0.99536	-0.25846
**b_2**	0	-2.03988	1.68419	-1.6385	-0.75325	-0.41678
**b_3**	0	0.30858	-0.2128	0.35523	0.25653	0.11096

Table S11. Virial fit parameters for C_2H_2 sorption on SIFSIX-22-Zn, TIFSIX-6-Zn, GEFSIX-4-Zn, SNFSIX-2-Zn, ZRFSIX-3-Zn and TAFSEVEN-1-Zn.

	SIFSIX-22-Zn	TIFSIX-6-Zn	GEFSIX-4-Zn	SNFSIX-2-Zn	ZRFSIX-3-Zn	TAFSEVEN-1-Zn
**Temperatures (K)**	273, 298	273, 298	273, 298	273, 298	273, 298	273, 298
**Adj. R^2**	0.99990	0.99996	0.99997	0.99993	0.99999	0.99998
**a_0**	-4303.15	-5566.6977	-5324.6302	-5113.08	-5161.7	-4842.63
**a_1**	-589.058	828.63302	-398.85753	770.4266	125.1755	119.3533
**a_2**	646.6441	-112.54044	298.13501	-172.653	123.8737	45.27715
**a_3**	-60.7595	17.14702	-40.74308	-3.93526	-63.6477	-20.7203
**a_4**	-0.70969	-0.22053	0.80018	12.08496	21.22658	7.83033
**a_5**	-0.15605	-0.01002	-0.07019	-0.96774	-1.91477	-0.66493
**b_0**	20.35144	24.66325	24.03879	23.07959	23.37142	23.20521
**b_1**	2.48466	-2.37565	1.61347	-2.33468	-7.75E-04	0.12066
**b_2**	-2.35711	0.36634	-0.98584	0.92304	-0.03926	-0.0767
**b_3**	0.27258	-0.04179	0.14074	-0.15454	-0.03845	-0.01539

Table S12. Virial fit parameters for C_2H_4 sorption on SIFSIX-22-Zn, TIFSIX-6-Zn, GEFSIX-4-Zn, SNFSIX-2-Zn, ZRFSIX-3-Zn and TAFSEVEN-1-Zn.

	SIFSIX-22-Zn	TIFSIX-6-Zn	GEFSIX-4-Zn	SNFSIX-2-Zn	ZRFSIX-3-Zn	TAFSEVEN-1-Zn
**Temperatures (K)**	273, 298	273, 298	273, 298	273, 298	273, 298	273, 298
**Adj. R^2**	0.99985	0.99994	0.99989	0.99981	0.99959	0.99972
**a_0**	-3980.01	-3800.48	-3793.36	-4017.65	-4004.53	-3932.4
**a_1**	-28.7335	-171.347	-83.2973	22.76589	-46.0234	91.63664
**a_2**	50.42547	80.9251	52.77757	41.65907	103.7589	66.3358
**a_3**	-23.7305	-26.0809	-19.0612	-11.7108	-27.8157	-19.6487
**a_4**	4.24715	4.13397	2.86731	3.03077	5.50389	3.18087
**a_5**	0	0	0	0	0	0
**b_0**	21.86565	21.19256	21.1054	21.93185	21.97471	21.77787
**b_1**	-3980.01	-3800.48	-3793.36	-4017.65	-4004.53	-3932.4
**b_2**	-28.7335	-171.347	-83.2973	22.76589	-46.0234	91.63664
**b_3**	50.42547	80.9251	52.77757	41.65907	103.7589	66.3358

Table S13. Virial fit parameters for C_2H_6 sorption on SIFSIX-22-Zn, TIFSIX-6-Zn, GEFSIX-4-Zn, SNFSIX-2-Zn, ZRFSIX-3-Zn and TAFSEVEN-1-Zn.

	SIFSIX-22-Zn	TIFSIX-6-Zn	GEFSIX-4-Zn	SNFSIX-2-Zn	ZRFSIX-3-Zn	TAFSEVEN-1-Zn
**Temperatures (K)**	273, 298	273, 298	273, 298	273, 298	273, 298	273, 298
**Adj. R^2**	0.9998	0.99995	0.99979	0.99985	0.99983	0.99985
**a_0**	-3912.5	-3712.42	-3729.68	-3913.63	-3927.05	-3791.01
**a_1**	-205.47	-250.36	-244.574	-35.7537	-140.691	-125.063
**a_2**	223.9648	126.1605	142.8398	116.8137	194.6892	112.4218
**a_3**	-92.0137	-43.1196	-48.1985	-41.4804	-89.3749	-35.3907

**a_4**	12.55258	6.18255	6.00821	6.45008	17.37054	5.46445
**a_5**	0	0	0	0	0	0
**b_0**	21.30495	20.99762	20.90752	21.70605	21.71159	21.29154
**b_1**	0.90428	0.91876	0.83062	0.33876	0.58142	0.55252
**b_2**	0	0	0	0	0	0
**b_3**	0	0	0	0	0	0

(a) IAST Selectivity Calculations

The selectivities for the adsorbate mixture composition of interest were calculated from the single-component adsorption isotherms using Ideal Adsorbed Solution Theory (IAST).[3, 4] Single-component adsorption isotherms for each gas at 298 K were fitted to the thermodynamically consistent dual-site Langmuir equation (Equation 3).

$$n(P) = \frac{q_1(k_1P)}{1+(k_1P)} + \frac{q_2(k_2P)}{1+(k_2P)} \quad \textbf{Eqn. 3}$$

In this equation, *P* is the total pressure (bar) of the bulk gas at equilibrium with the adsorbed phase, q_1 and q_2 are the saturation uptakes (in mmol g^{-1}) for sites 1 and 2 respectively, k_1 and k_2 are the affinity coefficients (in bar^{-1}) for sites 1 and 2 respectively, and $n(P)$ is the uptake (mmol g^{-1}) as a function of pressure. Once the isotherms have been parametrised, mixed-gas fractional uptakes are calculated, and finally the selectivity, $S_{i/j}$, is obtained using Equation 4. Here, x_i and x_j are the mole fractions of components *i* and *j*, respectively, in the adsorbed phase, and y_i and y_j are the mole fractions of components *i* and *j*, respectively, in the gas phase.

$$S_{i/j} = \frac{(x_i/x_j)}{(y_i/y_j)} \quad \textbf{Eqn. 4}$$

Dual-site Langmuir parameters for various equations are listed in Table S4.

Table S14. Isotherm fitting parameters and fit R^2 values for IAST calculations for SIFSIX-22-Zn.

Adsorbate	Model	R^2	q_1 (mmol g^{-1})	k_1 (bar^{-1})	q_2 (mmol g^{-1})	k_2 (bar^{-1})
**CO_2**	Dual-site Langmuir	0.999997	1.42123	36.3516	5.09202	1.24955
**C_2H_2**	Dual-site Langmuir	0.999993	3.79423	66.1418	3.24871	1.39499
**C_2H_4**	Dual-site Langmuir	0.999989	1.2831	13.9329	3.57155	1.02889

Table S15. Isotherm fitting parameters and fit R^2 values for IAST calculations for TIFSIX-6-Zn.

Adsorbate	Model	R^2	q_1 (mmol g^{-1})	k_1 (bar^{-1})	q_2 (mmol g^{-1})	k_2 (bar^{-1})
**CO_2**	Dual-site Langmuir	0.999895	0.990854	113.848	5.33402	1.63973
**C_2H_2**	Dual-site Langmuir	0.999971	3.34826	65.6454	3.02498	3.18497
**C_2H_4**	Dual-site Langmuir	0.999997	2.42572	8.65195	2.20176	1.11133

Table S16. Isotherm fitting parameters and fit R^2 values for IAST calculations for GEFSIX-4-Zn.

Adsorbate	Model	R^2	q_1 (mmol g^{-1})	k_1 (bar^{-1})	q_2 (mmol g^{-1})	k_2 (bar^{-1})
**CO_2**	Dual-site Langmuir	0.999916	1.15557	58.3329	5.04909	1.81579
**C_2H_2**	Dual-site Langmuir	0.999996	3.88062	52.1469	2.15275	2.43301
**C_2H_4**	Dual-site Langmuir	0.999998	2.16897	9.26804	3.33908	1.1745

Table S17. Isotherm fitting parameters and fit R^2 values for IAST calculations for SNFSIX-2-Zn.

Adsorbate	Model	R^2	q_1 (mmol g^{-1})	k_1 (bar^{-1})	q_2 (mmol g^{-1})	k_2 (bar^{-1})
**CO_2**	Dual-site Langmuir	0.999991	0.989799	86.6477	4.74835	1.59896
**C_2H_2**	Dual-site Langmuir	0.999926	2.8117	83.6293	2.58792	4.32093
**C_2H_4**	Dual-site Langmuir	0.999998	2.15028	9.42726	2.3089	0.836022

Table S18. Isotherm fitting parameters and fit R^2 values for IAST calculations for ZRFSIX-3-Zn.

Adsorbate	Model	R^2	q_1 (mmol g^{-1})	k_1 (bar^{-1})	q_2 (mmol g^{-1})	k_2 (bar^{-1})
**CO_2**	Dual-site Langmuir	0.999998	0.984434	136.813	4.33374	1.55391
**C_2H_2**	Dual-site Langmuir	0.999855	2.28435	84.5589	2.58799	5.73615
**C_2H_4**	Dual-site Langmuir	0.999986	2.85278	7.99839	171.328	0.0026463

Table S19. Isotherm fitting parameters and fit R^2 values for IAST calculations for TAFSEVEN-1-Zn.

Adsorbate	Model	R^2	q_1 (mmol g^{-1})	k_1 (bar^{-1})	q_2 (mmol g^{-1})	k_2 (bar^{-1})
**CO_2**	Dual-site Langmuir	0.999774	1.24341	30.4567	8.8263	0.358286
**C_2H_2**	Dual-site Langmuir	0.999944	1.61964	42.6036	3.64425	5.36697
**C_2H_4**	Dual-site Langmuir	0.999998	2.54006	7.4312	4.5193	0.283055

(a) Comparisons of Performance Parameters

Performance parameters of a number of leading hybrid coordination networks (HCNs) for gas separation applications are tabulated below (Table S20 – S22). Some hybrid ultramicroporous materials are known to show ultrahigh (sometimes >10^4) calculated selectivities, combined with relatively low uptakes (<2.5 mmol g^{-1}). These sorbents are omitted from the plots in the main manuscript (Fig. 3) in order to present a meaningful comparison.

Table S20. 1 bar uptakes of SIFSIX-22-Zn, TIFSIX-6-Zn, GEFSIX-4-Zn, SNFSIX-2-Zn, ZRFSIX-3-Zn and TAFSEVEN-1-Zn for various adsorbates at 298 K.

Compound	CO_2 uptake (1 bar, 298 K)) (mmol g^{-1})	C_2H_2 uptake (1 bar, 298 K)) (mmol g^{-1})	C_2H_4 uptake (1 bar, 298 K)) (mmol g^{-1})	C_2H_6 uptake (1 bar, 298 K)) (mmol g^{-1})
**SIFSIX-22-Zn**	4.2	5.7	3.0	2.9
**TIFSIX-6-Zn**	4.3	5.7	3.3	3.1
**GEFSIX-4-Zn**	4.4	5.4	3.8	3.4
**SNFSIX-2-Zn**	3.9	5.0	3.0	2.8
**ZRFSIX-3-Zn**	3.6	4.6	3.0	2.6
**TAFSEVEN-1-Zn**	3.5	4.7	3.2	2.9

Table S21. Performance parameters of leading C_2H_2/C_2H_4 selective HCNs.

Compound	S_{AE} (1:99)	C_2H_2 Q_{st} (kJ mol^{-1})	C_2H_2 uptake (mmol g^{-1})	Reference
**TIFSIX-14-Cu-i**	229	54	3.8	30
**GeFSIX-2-Cu-i**	67	42.6	3.9	31
**TIFSIX-2-Cu-i**	55	46	3.9	32
**SIFSIX-2-Cu-i**	44.5	52.9	4.02	33
**SIFSIX-1-Cu**	10.6	30	8.5	33
**UTSA-220**	10	29	3.4	34
**SIFSIX-3-Zn**	8.8	21	3.6	33
**SIFSIX-2-Cu**	6	26.3	5.4	33
**SIFSIX-3-Ni**	5	36.7	3.3	32
**ZJU-280**	44.5 (296 K)	51.0	4.74	30
**SIFSIX-22-Zn**	18.7	36.5	5.7	this work
**TIFSIX-6-Zn**	11.5	44.8	5.7	this work
**GEFSIX-4-Zn**	9.8	44.8	5.4	this work
**SNFSIX-2-Zn**	12.8	41.3	5.0	this work
**ZRFSIX-3-Zn**	8.7	42.7	4.6	this work
**TAFSEVEN-1-Zn**	4.7	40.0	4.7	this work

Table S22. Performance parameters of leading C_2H_2/CO_2 selective HCNs.

Compound	S_{AC} (1:1)	C_2H_2 Q_{st} (kJ mol^{-1})	C_2H_2 uptake (mmol g^{-1})	Reference
**Dicro-4-Ni-i**	18.2	37.7	1.9	35
**TIFSIX-2-Cu-i**	10	46	4.1	32
**UTSA-220**	4.4	29	3.4	34
**SIFSIX-3-Ni**	0.13	36.7	3.3	32
**ZJU-280**	18.1 (296 K)	51.0	4.74	50
**SIFSIX-22-Zn**	6.5	36.5	5.7	51
**TIFSIX-6-Zn**	5.3	44.8	5.7	this work
**GEFSIX-4-Zn**	5.0	44.8	5.4	this work
**SNFSIX-2-Zn**	5.6	41.3	5.0	this work
**ZRFSIX-3-Zn**	4.4	42.7	4.6	this work
**TAFSEVEN-1-Zn**	3.8	40.0	4.7	this work

References

1. Sarkisov, L. Poreblazer v4.0, University of Manchester, Manchester, **2020**.
2. R. S. Rowland and R. Taylor, *J. Phys. Chem.*, 1996, **100**, 7384-7391.
3. A. Bondi, *J. Phys. Chem.*, 1964, **68**, 441-451.
4. S. Subramanian and M. J. Zaworotko, *Angew. Chem. Int. Ed.*, 1995, **34**, 2127-2129.
5. S. i. Noro, S. Kitagawa, M. Kondo and K. Seki, *Angew. Chem. Int. Ed.*, 2000, **39**, 2081-2084.
6. P. Nugent, V. Rhodus, T. Pham, B. Tudor, K. Forrest, L. Wojtas, B. Space and M. Zaworotko, *Chem. Commun.*, 2013, **49**, 1606-1608.
7. M.-J. Lin, A. Jouaiti, N. Kyritsakas and M. W. Hosseini, *CrystEngComm*, 2009, **11**, 189-191.
8. P. Nugent, Y. Belmabkhout, S. D. Burd, A. J. Cairns, R. Luebke, K. Forrest, T. Pham, S. Ma, B. Space and L. Wojtas, *Nature*, 2013, **495**, 80-84.
9. K. Uemura, A. Maeda, T. K. Maji, P. Kanoo and H. Kita, *Eur. J. Inorg. Chem.*, 2009, 2329-2337.
10. O. Shekhah, Y. Belmabkhout, Z. Chen, V. Guillerm, A. Cairns, K. Adil and M. Eddaoudi, *Nat. commun.*, 2014, **5**, 1-7.
11. M.-J. Lin, A. Jouaiti, N. Kyritsakas and M. W. Hosseini, *CrystEngComm*, 2011, **13**, 776-778.
12. S. D. Burd, S. Ma, J. A. Perman, B. J. Sikora, R. Q. Snurr, P. K. Thallapally, J. Tian, L. Wojtas and M. J. Zaworotko, *J. Am. Chem. Soc.*, 2012, **134**, 3663-3666.
13. S. Xiong, Y. He, R. Krishna, B. Chen and Z. Wang, *Cryst. Growth Des.*, 2013, **13**, 2670-2674.
14. J.-J. Liu, Y.-J. Hong, Y.-F. Guan, M.-J. Lin, C.-C. Huang and W.-X. Dai, *Dalton Trans.*, 2015, **44**, 653-658.
15. D. Sensharma, S. Vaesen, C. Healy, J. Hartmann, A. C. Kathalikkattil, P. Wix, F. Steuber, N. Zhu and W. Schmitt, *Eur. J. Inorg. Chem.*, 2018, 1993-1997.
16. A. Bajpai, M. Lusi and M. J. Zaworotko, *Chem. Commun.*, 2017, **53**, 3978-3981.
17. V. V. Ponomarova, V. V. Komarchuk, I. Boldog, H. Krautscheid and K. V. Domasevitch, *CrystEngComm*, 2013, **15**, 8280-8287.
18. W. Liang, P. M. Bhatt, A. Shkurenko, K. Adil, G. Mouchaham, H. Aggarwal, A. Mallick, A. Jamal, Y. Belmabkhout and M. Eddaoudi, *Chem*, 2019, **5**, 950-963.
19. M. H. Mohamed, S. K. Elsaidi, L. Wojtas, T. Pham, K. A. Forrest, B. Tudor, B. Space and M. J. Zaworotko, *J. Am. Chem. Soc.*, 2012, **134**, 19556-19559.

20. R. Gautier, M. D. Donakowski and K. R. Poeppelmeier, *J.Solid State Chem.*, 2012, **195**, 132-139.
21. A. Cadiau, K. Adil, P. Bhatt, Y. Belmabkhout and M. Eddaoudi, *Science*, 2016, **353**, 137-140.
22. A. Cadiau, Y. Belmabkhout, K. Adil, P. M. Bhatt, R. S. Pillai, A. Shkurenko, C. Martineau-Corcos, G. Maurin and M. Eddaoudi, *Science*, 2017, **356**, 731-735.
23. H. S. Scott, A. Bajpai, K.-J. Chen, T. Pham, B. Space, J. J. Perry and M. J. Zaworotko, *Chem. Commun.*, 2015, **51**, 14832-14835.
24. H. S. Scott, M. Shivanna, A. Bajpai, K.-J. Chen, D. G. Madden, J. J. Perry IV and M. J. Zaworotko, *Cryst. Growth Des.*, 2017, **17**, 1933-1937.
25. M. Lusi, P. B. Fechine, K.-J. Chen, J. J. Perry and M. J. Zaworotko, *Chem. Commun.*, 2016, **52**, 4160-4162.
26. S. K. Elsaidi, M. H. Mohamed, T. Pham, T. Hussein, L. Wojtas, M. J. Zaworotko and B. Space, *Cryst. Growth Des.*, 2016, **16**, 1071-1080.
27. Q. Lin, C. Mao, A. Kong, X. Bu, X. Zhao and P. Feng, *J. Mat. Chem. A*, 2017, **5**, 21189-21195.
28. A. L. Myers and J. M. Prausnitz, *AIChE J.*, 1965, **11**, 121-127.
29. K. S. Walton and D. S. Sholl, *AIChE J.*, 2015, **61**, 2757-2762.
30. D. O'Nolan, A. Kumar, K.-J. Chen, S. Mukherjee, D. G. Madden and M. J. Zaworotko, *ACS Appl. Nano Mater.*, 2018, **1**, 6000-6004.
31. Z. Zhang, X. Cui, L. Yang, J. Cui, Z. Bao, Q. Yang and H. Xing, *Ind. Eng. Chem. Res.*, 2018, **57**, 7266-7274.
32. K.-J. Chen, H. S. Scott, D. G. Madden, T. Pham, A. Kumar, A. Bajpai, M. Lusi, K. A. Forrest, B. Space and J. J. Perry IV, *Chem*, 2016, **1**, 753-765.
33. X. Cui, K. Chen, H. Xing, Q. Yang, R. Krishna, Z. Bao, H. Wu, W. Zhou, X. Dong and Y. Han, *Science*, 2016, **353**, 141-144.
34. H. Li, L. Li, R.-B. Lin, G. Ramirez, W. Zhou, R. Krishna, Z. Zhang, S. Xiang and B. Chen, *ACS Sust. Chem. Eng.*, 2019, **7**, 4897-4902.
35. H. S. Scott, M. Shivanna, A. Bajpai, D. G. Madden, K.-J. Chen, T. Pham, K. A. Forrest, A. Hogan, B. Space and J. J. Perry IV, *ACS Appl. Mater. Interfaces*, 2017, **9**, 33395-33400.
36. P. M. Bhatt, Y. Belmabkhout, A. Cadiau, K. Adil, O. Shekhah, A. Shkurenko, L. J. Barbour and M. Eddaoudi, *J. Am. Chem. Soc.*, 2016, **138**, 9301-9307.
37. A. Kumar, C. Hua, D. G. Madden, D. O'Nolan, K.-J. Chen, L.-A. J. Keane, J. J. Perry and M. J. Zaworotko, *Chem. Commun.*, 2017, **53**, 5946-5949.
38. H. S. Scott, N. Ogiwara, K.-J. Chen, D. G. Madden, T. Pham, K. Forrest, B. Space, S. Horike, J. J. Perry IV and S. Kitagawa, *Chem. Sci.*, 2016, **7**, 5470-5476.
39. S. Mukherjee, N. Sikdar, D. O'Nolan, D. M. Franz, V. Gascón, A. Kumar, N. Kumar, H. S. Scott, D. G. Madden and P. E. Kruger, *Science advances*, 2019, **5**, eaax9171.
40. A. K. Rappé, C. J. Casewit, K. Colwell, W. A. Goddard III and W. M. Skiff, *J. Am. Chem. Soc.*, 1992, **114**, 10024-10035.
41. P. P. Ewald, *Annalen der Physik*, 1921, **369**, 253-287.
42. B. A. Wells and A. L. Chaffee, *J. Chem. Theory Comput.*, 2015, **11**, 3684-3695.
43. G. Kresse and J. Furthmüller, *Comput. Mater. Sci.*, 1996, **6**, 15-50.
44. G. Kresse and J. Furthmüller, *Phys. **Rev.** B.*, 1996, **54**, 11169.
45. J. Wellendorff, K. T. Lundgaard, A. Møgelhøj, V. Petzold, D. D. Landis, J. K. Nørskov, T. Bligaard and K. W. Jacobsen, *Physical Review B*, 2012, **85**, 235149.
46. P. E. Blöchl, *Phys. Rev. B.*, 1994, **50**, 17953.
47. J. D. Pack and H. J. Monkhorst, *Phys. Rev. B.*, 1977, **16**, 1748.
48. H. J. Monkhorst and J. D. Pack, *Phys. Rev. B.*, 1976, **13**, 5188.
49. A. Ghysels, T. Verstraelen, K. Hemelsoet, M. Waroquier and V. Van Speybroeck, *J. Chem. Inf. Model.*, 2010, **50**, 1736-1750.

50. Q.-L. Qian, X.-W. Gu, J. Pei, H.-M. Wen, H. Wu, W. Zhou, B. Li and G. Qian, *J. Mater. Chem. A*, **2021**, *9*, 9248-9255.

51. D. Sensharma, D. O'Hearn, A. Koochaki, A. Bezrukov, N. Kumar, B. Wilson, M. Vandichel, and M. Zaworotko, *Angew. Chem. Int. Ed.,* 2022, **61**, e202116145.
